# Disitamab Vedotin Plus Tislelizumab in Transurethrally Unresectable, *ERBB2*-Positive, Non–Muscle-Invasive Bladder Cancer: A Nonrandomized Clinical Trial

**DOI:** 10.1001/jamaoncol.2026.3909

**Published:** 2026-10-08

**Authors:** Yunkai Qie, Kaipeng Jia, Shiwang Huang, Jiazeng Zhao, Zihan Xue, Shizheng Guo, Tianxiao Zhang, Zhouliang Wu, Chong Shen, Hailong Hu

**Affiliations:** 1 Department of Urology, Tianjin Institute of Urology, The Second Hospital of Tianjin Medical University, Tianjin, China; 2 Tianjin Key Laboratory of Urology, Tianjin Institute of Urology, The Second Hospital of Tianjin Medical University, Tianjin, China; 3 Department of Urology Surgery, Shanxi Provincial People’s Hospital Affiliated to Shanxi Medical University, Taiyuan, Shanxi, China; 4 Department of Urology, The Third Medical Center, Chinese People’s Liberation Army General Hospital, Beijing, China; 5 Department of Urology, Chu Hsien-I Memorial Hospital, Tianjin Medical University, Tianjin, China

## Abstract

**Importance:**

Very high-risk non–muscle-invasive bladder cancer (VHR NMIBC) includes extensive high-grade T1 (T1HG) disease, T1HG with carcinoma in situ, variant histology, lymphovascular invasion, or bacille Calmette-Guérin (BCG)–unresponsive disease. Transurethrally unresectable disease warrants radical cystectomy, but patients ineligible for or declining cystectomy lack established nonsurgical therapy.

**Objective:**

To evaluate the efficacy and safety of disitamab vedotin (DV, an anti-*ERBB2* [formerly *HER2*] antibody-drug conjugate) plus tislelizumab (an anti–programmed cell death 1 protein immune checkpoint inhibitor) in transurethrally unresectable *ERBB2*-positive VHR NMIBC.

**Design, Setting, and Participants:**

This was an open-label, single-arm, phase 2, nonrandomized clinical trial with a Simon 2-stage design, conducted in an academic hospital from August 2022 to May 2024 with a median follow-up of 29.4 months. The analysis included adults with transurethrally unresectable *ERBB2*-positive NMIBC who met the 2019 European Association of Urology VHR criteria and who were ineligible for or declined cystectomy.

**Intervention:**

Patients received an intravenous injection of DV (120 mg, day 1) plus tislelizumab (200 mg, day 2) every 3 weeks for 3 cycles; patients who responded received up to 8 DV cycles and 17 tislelizumab cycles.

**Main Outcomes and Measures:**

The primary outcome was complete response (CR) of high-risk disease, defined as no high-grade tumor, T1 disease, carcinoma in situ, or progression. Secondary outcomes were response duration, progression-free survival, overall survival, and safety.

**Results:**

Among 137 screened patients, 28 participants (median [IQR] age, 72 [67-74] years; 25 male [89%]) were included. Two preassessment withdrawals were counted as nonresponders in the all-treated analysis, which left 26 efficacy-evaluable patients. CR of high-risk disease was achieved in 19 of 26 (73.1%; 95% CI, 54.8%-91.3%), meeting the prespecified 70% alternative-rate goal. Among all 28, 19 responded (67.9%; 95% CI, 49.4%-86.3%), exceeding the 17-response exact binomial threshold. Both analyses met the primary criterion. Among responders, the 24-month sustained response was 83.5% (95% CI, 68.0%-100.0%), and no muscle invasive or metastatic progression occurred. Treatment-related adverse events occurred in 25 patients (89.3%), most commonly paresthesia (12 [42.9%]), alopecia (11 [39.3%]), and pruritus (10 [35.7%]); 4 (14.3%) had grade 3 to 4 events; 2 (7.1%) discontinued treatment; and none had grade 5 events.

**Conclusions and Relevance:**

Findings of this nonrandomized clinical trial suggest that DV plus tislelizumab may show a high CR rate and durable disease control in patients with transurethrally unresectable *ERBB2*-positive VHR NMIBC, supporting randomized clinical trials.

**Trial Registration:**

ClinicalTrials.gov Identifier: NCT05495724

## Key Points

**Question:**

What are the outcomes of disitamab vedotin plus tislelizumab for transurethrally unresectable, *ERBB2 *(formerly *HER2*)–positive, very high-risk, non–muscle-invasive bladder cancer (VHR NMIBC)?

**Findings:**

In this single-arm, phase 2, nonrandomized clinical trial including 28 adults, more than half of efficacy-evaluable patients with 2019 European Association of Urology–defined VHR NMIBC achieved complete response (CR) of high-risk disease, meeting the alternative-rate goal, and a similar number exceeded the 17-response threshold in the all-treated analysis. The 24-month sustained response was high, with some grade 3 to 4 treatment-related adverse events reported.

**Meaning:**

Results suggest that disitamab vedotin plus tislelizumab provided a high CR rate and durable disease control in patients with transurethrally unresectable *ERBB2*-positive VHR NMIBC, and further evaluation of this investigational regimen in randomized clinical trials is recommended.

## Teaser

This nonrandomized clinical trial examines the efficacy of disitamab vedotin plus tislelizumab for transurethrally unresectable, *ERBB2*-positive, very high-risk, non–muscle-invasive bladder cancer in adults who were ineligible for or declined radical cystectomy.

## Introduction

Non–muscle-invasive bladder cancer (NMIBC) is commonly managed with complete transurethral resection of bladder tumor (TURBT) followed by risk-adapted intravesical therapy.^1^ Very high-risk (VHR) NMIBC is a guideline-defined risk category that includes features such as extensive high-grade T1 (T1HG) disease, T1HG with carcinoma in situ (CIS), variant histology, lymphovascular invasion, or bacille Calmette-Guérin (BCG)–unresponsive disease.^3^ Patients with transurethrally unresectable VHR NMIBC are generally recommended to undergo immediate radical cystectomy when feasible.^3^ More than 60% of patients with VHR NMIBC are medically ineligible for upfront radical cystectomy or decline the procedure,^4^ and for these patients established nonsurgical treatment options are limited.

In the previous phase 2 TRUCE-02 trial (NCT04730232), tislelizumab plus low-dose nab-paclitaxel achieved a 62.7% complete response (CR) rate of high-risk disease in patients with transurethrally unresectable VHR NMIBC,^5^ establishing proof of concept that systemic therapy could achieve meaningful disease clearance in this setting. Tislelizumab is an anti–programmed cell death 1 protein (PD-1) antibody that restores antitumor immune responses by blocking PD-1–mediated immune suppression. Building on these findings, the emergence of *ERBB2 *(formerly* HER2*)–directed antibody-drug conjugates (ADCs) provided an opportunity to further develop biomarker-selected systemic therapy in this setting. *ERBB2* positivity is reported in approximately 40% to 50% of bladder cancers.^6^ Disitamab vedotin (DV) is an *ERBB2*-targeted ADC that delivers a cytotoxic payload to *ERBB2*-expressing tumor cells, and DV combined with a PD-1 blockade has shown promising clinical activity in advanced urothelial carcinoma.^8^ Together, the biologic complementarity and clinical activity observed in advanced disease provided the rationale for evaluating DV plus tislelizumab in patients with transurethrally unresectable *ERBB2*-positive VHR NMIBC.

In this study, we describe the results of the single-arm, phase 2 TRUCE-04 trial, in which we evaluated disitamab vedotin plus tislelizumab in patients with transurethrally unresectable *ERBB2*-positive VHR NMIBC. We hypothesized that this regimen could clear residual high-risk disease in 70% of patients, informed by prior response rates of 56% to 75%.^10^

## Methods

This nonrandomized clinical trial was reviewed and approved by the institutional review board of the Second Hospital of Tianjin Medical University and conducted in accordance with the Declaration of Helsinki and International Conference on Harmonization Good Clinical Practice guidelines. All patients provided written informed consent. The study followed the Transparent Reporting of Evaluations With Nonrandomized Designs (TREND) reporting guidelines.

### Patients

The TRUCE-04 trial was developed after the phase 2 TRUCE-02 trial and used the same core eligibility criteria for transurethrally unresectable VHR NMIBC, with the addition of *ERBB2* expression requirements. Race and ethnicity data were collected in routine demographic documentation and were available from the participants’ CRFs and medical records, although these variables were not separately prespecified for subgroup analyses in the protocol. Patients were assessed for eligibility from August 2022 to May 2024; eligible participants were adults diagnosed with transurethrally unresectable VHR NMIBC who were ineligible for or declined radical cystectomy. The definition of transurethrally unresectable bladder tumor required visually incomplete transurethral resection or high-volume tumor, confirmed by cystoscopy, multipoint bladder biopsies, and independent verification by at least 2 senior urologists. High-volume tumors were defined as single or confluent lesions greater than 5 cm in diameter or multifocal lesions accounting for greater than 30% of the total bladder surface area.^5^ Pre-enrollment screening for VHR NMIBC followed the 2019 European Association of Urology criteria as follows: encompassing multiple, large, or recurrent T1HG tumors; T1HG with concurrent bladder CIS; T1HG with CIS in the prostatic urethra; variant histology of urothelial carcinoma; lymphovascular invasion; or BCG-unresponsive disease.^3^

Additional key eligibility criteria included histologically confirmed urothelial carcinoma (dominant component ≥50% for mixed histology), *ERBB2*-positive (immunohistochemistry [IHC] 2+ or 3+), Eastern Cooperative Oncology Group (ECOG) performance status 0 to 2, adequate organ function, and positive postoperative urine cytology within 12 weeks of diagnostic TURBT. The detailed inclusion and exclusion criteria are provided in the trial protocol in Supplement 1.

### Study Design

The TRUCE-04 trial was an open-label, single-arm, phase 2 pilot study evaluating the efficacy of DV combined with tislelizumab in patients with *ERBB2*-positive VHR NMIBC. The primary end point was the CR rate of high-risk disease, defined as the absence of T1 tumors, high-grade tumors, CIS, and progressive disease (assessed approximately 3 months after initial treatment). Residual low-grade Ta disease was permitted under this end point definition. This end point was selected because all enrolled patients had residual, active, high-risk disease at baseline for which complete TURBT was not feasible. In this setting, clearance of high-risk disease after initial treatment provided an early measure of antitumor activity. Secondary end points included safety (treatment-related adverse events [TRAEs], graded per the National Cancer Institute Common Terminology Criteria for Adverse Events v5.0); CR rate of any disease (absence of low-grade Ta tumors, high-risk disease, and progressive disease); duration of CR (DOR) for high-risk disease and any disease (from initial CR to recurrence or progression of the corresponding disease); 24-month DOR rates; progression-free survival (PFS; enrollment to T-stage upstaging, metastasis, or death from any cause, whichever occurred first); 24-month PFS rate; overall survival (OS; enrollment to death); 24-month OS rate; and subsequent treatment outcomes.

### Procedures and Treatments

Treatment administration and assessment schedules were consistent with TRUCE-02,^5^ with modification of the chemotherapeutic agent. Patients received intravenous DV, 120 mg, on day 1 and intravenous tislelizumab, 200 mg, on day 2, repeated every 3 weeks as 1 treatment cycle for a total of 3 cycles initially. Comprehensive efficacy assessment was performed approximately 3 months after initial treatment, including TURBT biopsy (directed biopsies of original tumor sites and identified anomalies, plus random multipoint bladder biopsies and prostatic urethra biopsy for male patients if indicated), urine cytology, and imaging examination.

Patients achieving CR of high-risk disease continued treatment for up to 8 cycles of DV (approximately 6 months) and 17 cycles (1 year) of tislelizumab, or until disease recurrence, progression, unacceptable toxicity, or consent withdrawal. Patients who did not achieve CR of high-risk disease discontinued investigational treatment and were followed up for disease progression. Long-term follow-up included urine cytology and cystoscopy every 3 months for 2 years (then every 6 months for 3 years, with directed biopsies for abnormal cystoscopy findings and random biopsies for abnormal urine cytology) and chest- and pelvic-computed tomography every 6 months for 2 years (then annually for 3 years). Safety monitoring was conducted throughout treatment and follow-up. The data cut-off date was December 31, 2025.

### *ERBB2* Expression Assessment

*ERBB2* IHC staining was performed on the Ventana platform using the anti-ERBB2 (4B5) rabbit monoclonal antibody and the ultraView Universal DAB Detection Kit (Roche). Staining results were classified as IHC 0, 1+, 2+, or 3+, according to breast cancer criteria, with IHC 2+ and 3+ defined as *ERBB2* positive. Patients with *ERBB2*-positive tumors were eligible for enrollment, without requiring fluorescence in situ hybridization confirmation.

### Statistical Analysis

The study adopted a Simon 2-stage design with a 1-sided significance level of 5% and 80% statistical power. The null hypothesis assumed a CR rate of high-risk disease of 41% (based on prior studies of immune checkpoint inhibitors [ICIs] as monotherapy in BCG-unresponsive disease within the VHR NMIBC subgroup^12^). The alternative hypothesis assumed a CR rate of high-risk disease of 70%. This target was anchored to the 56% CR rate reported in the 2022 TRUCE-02 meeting abstract,^10^ which was available when the TRUCE-04 trial was designed; the study later reported a CR rate of 62.7%.^5^ Additional contemporaneous support came from the 2022 RC48-C014 study meeting abstract, which reported a 75% confirmed investigator-assessed objective response rate (ORR).^11^ The full published study provided mature regimen-level context.^9^ Because disease stage and response end points differed, the advanced-disease ORR was not used as a direct historical benchmark. Initially, 7 patients were enrolled; if more than 3 achieved CR of high-risk disease, an additional 16 patients would be enrolled. The study would be considered positive if at least 14 of the planned 23 efficacy-evaluable patients achieved CR of high-risk disease.

Continuous variables are presented as median (IQR) and categorical variables as number (%). CR rates and their 95% CIs were calculated using the Clopper-Pearson exact method. DOR, PFS, and OS were estimated using Kaplan-Meier curves. Analyses stratified by *ERBB2* IHC status and other subgroups were descriptive only. Sensitivity analyses used exact binomial tests in the efficacy analysis set (EAS) and all-treated population, with preassessment withdrawals counted as nonresponders in the latter. For 28 treated patients, 17 responses were required to reject the 41% null rate at a 1-sided significance level of *P* < .05; *P* values for other tests were 2-sided, and *P* < .05 was considered statistically significant. All statistical analyses were performed using R, version 4.2.2 (R Foundation).

## Results

### Study Participants

Between August 2022 and May 2024, 137 patients were assessed for eligibility; 109 were excluded before enrollment for not meeting prespecified eligibility criteria (Figure 1). Twenty-eight patients were consecutively enrolled, received at least 1 dose of study treatment, and constituted the safety analysis set. Of these, 26 completed the initial efficacy assessment and constituted the EAS. The remaining 2 patients were enrolled during the second stage of the Simon 2-stage design. Each received 1 treatment cycle and voluntarily withdrew consent approximately 1 month after enrollment; neither withdrawal was due to disease progression or treatment-related toxicity (Figure 1).

**Figure 1.**
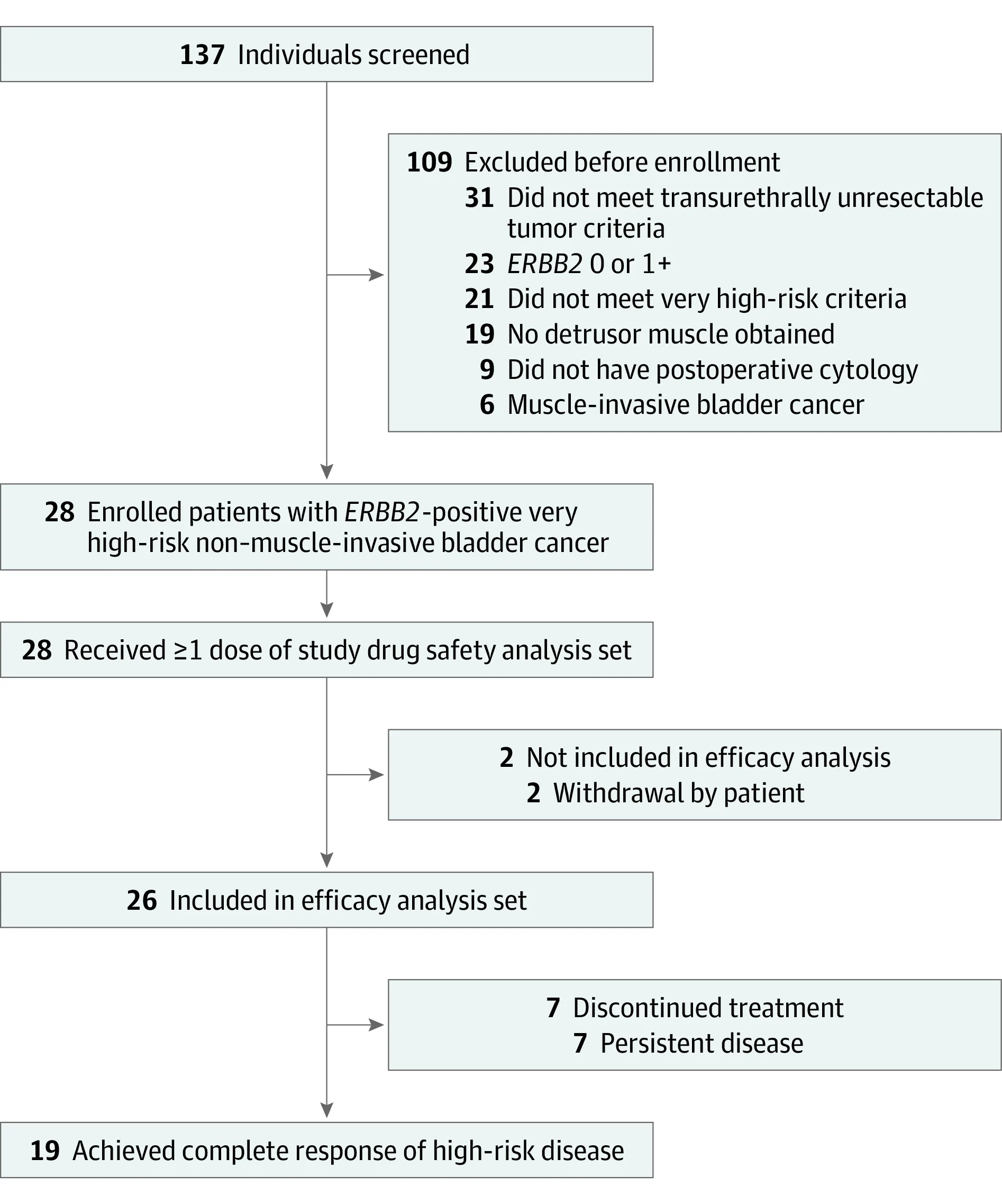
Flowchart of the Trial Among 137 screened patients, 28 patients with *ERBB2 *(formerly *HER2*)–positive, very high-risk, non–muscle-invasive bladder cancer received study treatment. Twenty-six patients completed the initial efficacy assessment; 2 voluntarily withdrew consent after 1 treatment cycle and before the initial assessment and were counted as nonresponders in the all-treated analysis. Complete response of high-risk disease was achieved in 19 patients.

Among the 28 enrolled patients, 3 (11%) were female and 25 (89%) were male; the median age was 72 (IQR, 67-74) years. All enrolled patients were of Han Chinese ethnicity and were Chinese nationals (28 [100%]). *ERBB2* IHC 2+ and 3+ expression was observed in 15 patients (54%) and 13 patients (46%), respectively. The median follow-up duration was 29.4 (IQR, 24.2-43.6) months as of the data cutoff date (December 31, 2025). The median treatment duration was 6 (IQR, 2.1-12.4) months, with a median of 7 (IQR, 3-11) cycles administered. Baseline characteristics of the study population are summarized in Table 1.

**Table 1.** Patient Demographics and Baseline Characteristics (N = 28)

Characteristic	Patients, No. (%)
Age, median (IQR), y	72 (67-74)
Race	
Asian	28 (100%)
Ethnicity	
Han Chinese	28 (100%)
Sex	
Female	3 (11)
Male	25 (89)
Current or former smoker	11 (39)
ECOG performance status	
0	22 (79)
1	6 (21)
Patient type	
Has primary tumor	23 (82)
Has recurrent tumor	5 (18)
Received intravesical chemotherapy	3 (11)
Was BCG-unresponsive	2 (7)
Tumor stage	
T1HG alone	18 (64)
T1HG with CIS	8 (29)
CIS	2 (7)
Histology^a^	
Pure urothelial carcinoma	23 (82)
Micropapillary urothelial carcinoma	3 (11)
Microcystic urothelial carcinoma	1 (4)
Urothelial carcinoma with glandular differentiation	1 (4)
*ERBB2* status	
2+	15 (54)
3+	13 (46)

Abbreviations: BCG, bacille Calmette-Guérin; CIS, carcinoma in situ; ECOG, Eastern Cooperative Oncology Group; T1HG, high-grade T1.

^a^ Percentages may not sum to 100% due to rounding.

### Efficacy

#### Interim Analysis Results

After 7 enrolled patients completed the first stage, 5 patients (71.4%; 95% CI, 35.9%-93.7%) achieved CR of high-risk disease, exceeding the prespecified threshold of 4 responses required to proceed to the second stage.

#### Final Efficacy Results

Among the 26 patients in the EAS, 19 achieved a CR of high-risk disease rate of 73.1% (95% CI, 54.8%-91.3%), meeting the prespecified alternative-rate goal of 70% (Table 2). In the conservative analysis of all 28 treated patients, with the 2 preassessment withdrawals counted as nonresponders, the CR rate was 67.9% (95% CI, 49.4%-86.3%). The 19 observed responses exceeded the corresponding 17-response exact-binomial threshold; thus, although the point estimate was numerically below the 70% alternative response rate, the primary efficacy criterion was met in both analyses. Among these 19 patients, 17 had no residual disease at the initial assessment, and 2 had residual low-grade Ta disease only. The median number of treatment cycles before the initial response assessment was 3 (range, 3-5) cycles.

**Table 2.** Complete Response Rates of High-Risk Disease in the Efficacy Analysis Set and All-Treated Patients

End point	No.	% (95% CI)
All patients (n = 26)^a^		
Complete response	19	73 (55-91)
Nonresponders^b^	7	27 (9-45)
*ERBB2* IHC 2+ (n = 14)		
Complete response	11	79 (54-100)
Nonresponders^b^	3	21 (0-46)
*ERBB2* IHC 3+ (n = 12)		
Complete response	8	67 (35-98)
Nonresponders^b^	4	33 (2-65)
All-treated patients, conservative analysis (n = 28)^c^		
Complete response	19	68 (49-86)
Nonresponders^b^	9	32 (14-51)

Abbreviation: IHC, immunohistochemistry.

^a^ The efficacy analysis set comprised 26 patients; 2 patients were excluded from efficacy evaluation due to withdrawal before the initial assessment.

^b^ Defined as the continued presence of non–muscle-invasive bladder cancer without evidence of progression in tumor grade or an increase in the depth of invasion.

^c^ The conservative all-treated analysis included all 28 treated patients; the 2 patients who withdrew during stage 2 after 1 treatment cycle and before the initial efficacy assessment were counted as nonresponders.

The median DOR of high-risk disease was not reached. The 12-month and 24-month sustained response rates were 89.5% (95% CI, 76.7%-100.0%). and 83.5% (95% CI, 68.0%-100.0%), respectively (Figure 2A). A swimmer plot summarizes the timing and duration of CR of high-risk disease (Figure 2B). CR of high-risk disease rates across predefined subgroups are summarized descriptively in eFigure 1 in Supplement 2. CR of high-risk disease rates were 78.6% (95% CI, 54.0%-100.0%) in patients with *ERBB2* IHC 2+ and 66.7% (95% CI, 35.4%-98.0%) in those with *ERBB2* IHC 3+ (Table 2).

**Figure 2.**
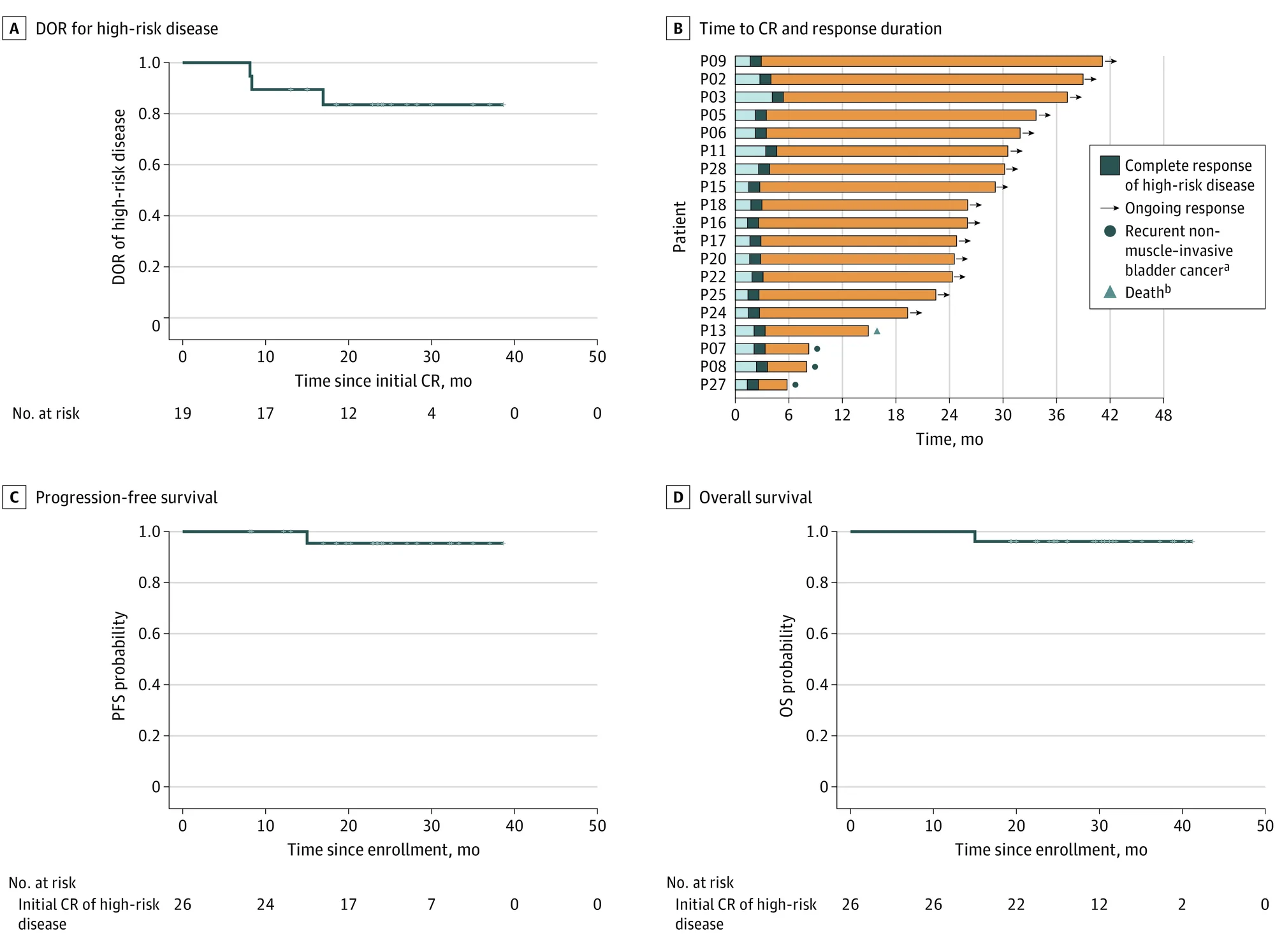
DOR and Survival Outcomes A, Line graph showing DOR for high-risk disease among the 19 patients who achieved an initial CR of high-risk disease. The sustained response rate was 89.5% (95% CI, 76.7-100) at 12 months and 83.5% (95% CI, 68.0-100) at 24 months. B, Swimmer plot illustrating time to CR of high-risk disease and response duration. C, Line graph showing progression-free survival. D, Line graph showing overall survival. CR indicates complete response; DOR, duration of response; OS, overall survival; PFS, progression-free survival. ^a^Recurrence was observed in 3 cases of T1 disease. ^b^One patient died of non–cancer-related causes (stroke).

For CR of any disease, 17 patients 65.4% (95% CI, 45.8%-85.0%) achieved CR (eTable 1 in Supplement 2). The median DOR was not reached, and the 12-month and 24-month sustained response rates were both 88.2% (95% CI, 74.2%-100.0%) (eTable 2 and eFigure 2 in Supplement 2). Among responders, DOR estimates stratified by *ERBB2* IHC status are summarized descriptively in eFigure 3 in Supplement 2.

The median PFS was not reached, with a 24-month PFS rate of 95.5% (95% CI, 87.1%-100.0%) (Figure 2C). No progression to muscle invasive or metastatic disease occurred. The median OS was not reached, with a 24-month OS rate of 96.2% (95% CI, 89.0%-100.0%) (Figure 2D). As of the data cutoff, only 1 death had been recorded—it was due to stroke and was unrelated to study treatment or disease progression. PFS and OS estimates stratified by *ERBB2* IHC status are summarized descriptively in eFigure 4 in Supplement 2.

### Safety

Among 28 patients in the safety analysis set, 25 (89.3%) experienced any-grade TRAEs, and 4 (14.3%) experienced grade 3 to 4 TRAEs. No treatment-related deaths or grade 5 adverse events (AEs) were reported. The most common any-grade TRAEs included paresthesia (12 [42.9%]), alopecia (11 [39.3%]), pruritus (10 [35.7%]), and rash (7 [25.0%]). Grade 3 to 4 TRAEs included fatigue (2 [7.1%]), rash (1 [3.6%]), hypopituitarism (1 [3.6%]), and hyperglycemia (1 [3.6%]) (Table 3). DV-related AEs occurred in 22 patients (78.6%), most commonly paresthesia (12 [42.9%]). No grade 3 or 4 DV-related AEs were observed (eTable 2 in Supplement 2). Immune-related AEs occurred in 17 patients (60.7%), most commonly pruritus (10 [35.7%]). Grade 3 to 4 immune-related AEs were observed in 4 patients (14.3%), most commonly fatigue (eTable 3 in Supplement 2). AEs were addressed with standard supportive care or temporary treatment interruption. Two patients (7.1%) discontinued treatment due to grade 3 to 4 TRAEs (1 due to rash and 1 due to hypopituitarism). No dose adjustments were required during the study.

**Table 3.** Common Treatment-Related Adverse Events (N = 28)

Adverse event	Patients, No. (%)			
	Any grade	Grade 1-2	Grade 3	Grade 4
Any	25 (89)	25 (89)	4 (14)	1 (4)
Paresthesia	12 (43)	12 (43)	0	0
Alopecia	11 (39)	11 (39)	0	0
Pruritus	10 (36)	10 (36)	0	0
Rash	7 (25)	6 (21)	1 (4)	0
Anorexia	6 (21)	6 (21)	0	0
Hypothyroidism	6 (21)	6 (21)	0	0
Fatigue	5 (18)	3 (11)	2 (7)	0
Hypopituitarism	5 (18)	3 (11)	1 (4)	1 (4)
Nausea	4 (14)	4 (14)	0	0
Hepatic dysfunction	2 (7)	2 (7)	0	0
Constipation	2 (7)	2 (7)	0	0
Dry mouth	2 (7)	2 (7)	0	0
Edema	1 (4)	1 (4)	0	0
Myalgia	1 (4)	1 (4)	0	0
Immune-mediated colitis	1 (4)	1 (4)	0	0
Hyperglycemia	1 (4)	0	1 (4)	0
Myocarditis	1 (4)	1 (4)	0	0
Increased creatine phosphokinase	1 (4)	1 (4)	0	0

### Subsequent Treatment

Among the 7 patients (26.9%) who did not achieve CR of high-risk disease, 4 (57.1%) underwent radical cystectomy. Of these, none had pathologic upstaging (classified as persistent disease at the initial assessment). Repeat *ERBB2* testing was performed on available posttreatment residual disease specimens. Baseline *ERBB2* status was IHC 2+ in 3 patients (42.9%) and IHC 3+ in 4 patients (57.1%); after treatment, 5 (71.4%) showed decreased *ERBB2* expression, including 4 conversions from *ERBB2*-overexpressing status (IHC 2+ or 3+) to low *ERBB2* expression (IHC 0 or 1+) (eFigure 5 in Supplement 2). No unexpected safety concerns were reported during radical cystectomy procedures (eTable 4 in Supplement 2).

Of the 19 patients (73.1%) who achieved CR of high-risk disease, 6 (31.6%) initiated subsequent therapeutic interventions following discontinuation of the study treatment as follows: 5 (26.3%) transitioned to intravesical instillation therapy, consisting of gemcitabine (n = 2) and BCG (n = 3); 1 patient (5.3%) continued systemic anticancer therapy; and 1 patient (5.3%) developed recurrent T1 disease, which was managed with intravesical BCG instillation.

## Discussion

Transurethrally unresectable VHR NMIBC represents a distinct clinical scenario in which complete TURBT followed by risk-adapted intravesical therapy is not feasible, leaving immediate radical cystectomy as the guideline-recommended approach for eligible patients. In this single-arm, phase 2, pilot trial, systemic *ERBB2*-targeted ADC therapy combined with PD-1 blockade showed preliminary antitumor activity in patients with *ERBB2*-positive, unresectable, high-risk tumors. The CR rate of high-risk disease was 73.1% in the EAS, meeting the prespecified 70% alternative-rate goal. In the conservative all-treated analysis, 19 of 28 patients (67.9%) responded, exceeding the 17-response exact binomial threshold; therefore, the primary efficacy criterion was met in both analyses. Among responders, the 24-month sustained response rate was 83.5%, and no progression to muscle invasive or metastatic disease was observed during follow-up. These findings support further evaluation of this strategy in larger studies.

Most contemporary trials incorporating systemic therapy in NMIBC enroll patients after complete TURBT of any visible papillary tumors, including BCG-unresponsive cohorts and BCG-naive high-risk cohorts.^12^ These studies primarily evaluate systemic therapy as an adjunct to local resection, targeting CIS, microscopic residual disease, or recurrence risk. *ERBB2*-directed ADC therapy is also being explored in NMIBC. The HERO (NCT07207824) and FORMULA-01 (ChiCTR2400080767) trials are evaluating intravenous DV plus BCG in patients with *ERBB2*-expressing high-risk NMIBC, whereas the RC48-C029 study (NCT06378242) is investigating intravesical disitamab vedotin in this setting. A retrospective analysis reported higher 12-month recurrence-free survival with adjuvant DV than with BCG in patients with *ERBB2*-overexpressing disease.^17^ The TRUCE-04 trial focused on a distinct treatment context in which patients had residual bulky or visually incompletely resected high-risk disease not amenable to complete TURBT. This distinction should be acknowledged when interpreting the primary end point and considering future trial design, particularly because no established comparator directly matches this population.

The observed TRAEs were consistent with the known safety profiles of DV- and tislelizumab-containing regimens.^5^ In the setting of non–muscle-invasive disease, where patients may have long-expected survival and require long-term treatment, the potential long-term and quality-of-life effects of systemic therapy warrant careful consideration.^18^ Neuropathy-associated and dermatologic events, although mostly mild to moderate, should be recognized when selecting patients, counseling them about treatment expectations, planning monitoring, and providing supportive care in future studies.

### Limitations

This study has some limitations. The single-arm design and small sample size limit the strength of the conclusions, particularly because the enrolled population included both BCG-naive and BCG-exposed patients. The absence of a control group precludes direct efficacy comparisons, although no established nonsurgical comparator directly matches this population. Complete response of high-risk disease was selected to assess early antitumor activity in patients with residual, active, high-risk disease, but this end point has not been validated as a surrogate for survival. Although DOR, PFS, and OS data support durability, the limited number of events precluded formal evaluation of associations between initial response and long-term outcomes. Despite these limitations, results of the current study suggest further evaluation of ADC-ICI combinations in this challenging clinical scenario and informs the design of future randomized clinical trials in this underserved patient population.

## Conclusions

Results of this nonrandomized clinical trial suggest antitumor activity of disitamab vedotin plus tislelizumab in patients with transurethrally unresectable, *ERBB2*-positive, very high-risk non–muscle-invasive bladder cancer who are ineligible for or decline radical cystectomy. The observed CR rate and durable disease control support further evaluation of this ADC-ICI combination therapy in larger, multicenter randomized clinical trials to assess its efficacy and long-term clinical benefit.

## Notes

Supplement 1.
Trial Protocol

Supplement 2. **eFigure 1. **Forest Plot of CR Rates for High-Risk Disease Across Different Subgroups **eFigure 2. **DOR Among the 17 Patients Who Achieved an Initial CR of Any Disease **eFigure 3. **DOR by *ERBB2* Expression Status **eFigure 4. **PFS and OS by *ERBB2 *Expression Status **eFigure 5. ***ERBB2* Expression Dynamics in Noncomplete Responders After Treatment **eTable 1. **CR Rate of Any Disease in Efficacy Assessment (n=26) **eTable 2. **DV-Related Adverse Events (n=28) **eTable 3. **Immune-Mediated Adverse Events (n=28) **eTable 4. **Pathological Staging at Time of Radical Cystectomy in Patients Who Discontinued Investigational Treatment by Maximum T Stage (n=4)

Supplement 3.
Data Sharing Statement
