# Supplementary material for: Disitamab Vedotin Plus Tislelizumab in Transurethrally Unresectable, ERBB2-Positive, Non–Muscle-Invasive Bladder Cancer: A Nonrandomized Clinical Trial
Source: JAMA Oncol. 2026 Oct 8:e263909. Online ahead of print. doi: 10.1001/jamaoncol.2026.3909 (PMC13650702; doi:10.1001/jamaoncol.2026.3909)
Supplement: Supplementary file 1 [file jamaoncol-e263909-s001.pdf]

Phase II Open-Label Clinical Study on the Efficacy and Safety of Disitamab Vedotin Combined with Tislelizumab in the Treatment of HER2-Positive Very High-Risk Non-Muscle Invasive Bladder Cancer (VHR-NMIBC) Unresectable by Transurethral Resection of Bladder Tumor (TUR-BT)

Study Drugs:

Disitamab Vedotin, Tislelizumab

Principal Investigator:

Dr. HaiLong Hu, MD

Second Hospital of Tianjin Medical University

23 Pingjiang Road, Jianshan Street, Hexi, Tianjin 300211,

P.R. China.

huhailong@tmu.edu.cn

Contents

Protocol Summary 4
1 Study Background 10
1.1 Introduction 10
1.2 Disease Background 12
1.3 Study Drug: Disitamab Vedotin 13
1.3.1 Mechanism of Action of Disitamab Vedotin 13
1.3.2 Clinical Study Results of Disitamab Vedotin 14
1.4 Research Progress of RC48-ADC in the Treatment of HER2-Positive Urothelial Carcinoma 15
2 Study Objectives 15
2.1 Primary Study Endpoint 15
2.2 Secondary Study Endpoints 16
3 Overall Study Design 16
4 Study Population 17
4.1 Inclusion Criteria 17
4.2 Exclusion Criteria 18
4.3 Criteria for Patient Withdrawal from the Study 19
5 Study Drugs and Treatment Regimen 19
5.1 Treatment Regimen 19
5.1.1 Disitamab Vedotin Treatment Regimen 19
5.1.2 Administration of Disitamab Vedotin 19
5.1.3 Administration of Tislelizumab 21
5.2 Dose Adjustment 22
5.2.1 Dose Adjustment of Tislelizumab 22
5.2.2 Dose Adjustment of Disitamab Vedotin 25
5.2.3 Management of Infusion Reactions Related to Disitamab Vedotin and Tislelizumab 25
5.3 Toxicity Management Principles 27
5.3.1 Toxicity Management Principles for RC48-ADC 27
5.3.2 Toxicity Management Principles for Immune Checkpoint Inhibitors 28
5.4 Prohibited Concomitant Therapies 29
6 Study Evaluation and Procedures 29
6.1 Screening Period 29
6.2 On-Treatment Visits 30
6.3 Post-Treatment Evaluation 30
6.4 Methods for Obtaining Tissues for Tumor Pathological Examination 31
6.5 Follow-Up After the Completion of Investigational Treatment 31
7 Efficacy Evaluation 32
7.1 Initial Efficacy Evaluation 32
7.2 Follow-up 32
7.3 Safety Evaluation 32
8 Safety Reporting and Adverse Event Management 32
8.1 Definition of Adverse Event (AE) 32

8.2 Definition of Serious Adverse Event (SAE) 33
8.3 Evaluation of Adverse Events 33
8.4 Documentation of Adverse Events 34
8.4.1 Time Window for AE Collection 34
8.4.2 Follow-Up of Adverse Events 34
8.4.3 Content of AE Documentation 35
8.5 Reporting of Serious Adverse Events (SAEs) 35
9 Statistical Considerations 35
9.1 Statistical Analysis Populations and Sample Size Estimation 35
9.2.1 General Statistical Analysis Methods 35
9.2.2 Baseline Characteristic Analysis 35
9.3.3 Efficacy Analysis 36
9.3.4 Safety Analysis 36
10 Ethics 36
10.1 Ethics Committee (EC) 36
10.2 Ethics of the Study 36
10.3 Subject Information and Informed Consent 37
10.4 Protection of Subject Data 37
11 Study Management 37
11.1 Data Processing and Record Keeping 37
11.2 Protocol Amendment 38

90 **Protocol Summary**

Study Title	Phase II Open-Label Clinical Study on the Efficacy and Safety of Disitamab Vedotin Combined with Tislelizumab in the Treatment of HER2-Positive Very High-Risk Non-Muscle Invasive Bladder Cancer (VHR-NMIBC) Unresectable by Transurethral Resection of Bladder Tumor (TUR-BT)
Study Drugs	Disitamab Vedotin, Tislelizumab
Study Objectives	To evaluate the efficacy and safety of Disitamab Vedotin combined with Tislelizumab in the treatment of HER2-positive urothelial carcinoma.<br>**Primary Endpoint**:<br>• Evaluate the Complete Response Rate of High-Risk Disease (HR-CRR) of Disitamab Vedotin combined with Tislelizumab in the treatment of HER2-positive VHR-NMIBC unresectable by TUR-BT.<br>**Secondary Endpoints**:<br>• Evaluate the Complete Response Rate of Any Disease (CRR) of Disitamab Vedotin combined with Tislelizumab in the treatment of HER2-positive VHR-NMIBC unresectable by TUR-BT.<br>• Evaluate the Duration of Complete Response (DOR) of Any Disease and High-Risk Disease of Disitamab Vedotin combined with Tislelizumab in the treatment of HER2-positive VHR-NMIBC unresectable by TUR-BT.<br>• Evaluate the Progression-Free Survival (PFS) and Overall Survival (OS) of Disitamab Vedotin combined with Tislelizumab in the treatment of HER2-positive VHR-NMIBC unresectable by TUR-BT.<br>• Evaluate the safety, incidence of Serious Adverse Events (SAEs), and incidence of Treatment-Related Adverse Events (TRAEs) of Disitamab Vedotin combined with Tislelizumab (Adverse events are graded according to the Common Terminology Criteria for

	Adverse Events (CTCAE) Version 5.0).
Study Design	This is a prospective observational clinical study designed to evaluate the efficacy and safety of Disitamab Vedotin combined with Tislelizumab in the treatment of HER2-positive very high-risk non-muscle invasive bladder cancer (VHR-NMIBC) unresectable by transurethral resection of bladder tumor (TUR-BT).<br>Adverse events will be monitored during the study and graded according to the National Cancer Institute (NCI) Common Terminology Criteria for Adverse Events (CTCAE) Version 5.0.<br>All eligible patients meeting the inclusion and exclusion criteria will be invited to participate in the study. Patients will be considered in the study unless they die, withdraw from the study, are lost to follow-up, or the study is terminated. Patients are not required to make special visits or undergo evaluations for the study, and evaluations will be conducted by investigators based on clinical practice.<br>As an observational study, the diagnosis and treatment

	procedures listed in the protocol are clinical recommended procedures and not mandatory. The treatment and clinical visit schedule of enrolled patients will be determined by clinicians based on routine practice. Subject information, including efficacy and safety data, will be collected in accordance with routine diagnosis and treatment procedures. Although the protocol does not mandate clinical visits, for patients who have not been seen for at least 6 months, investigators are encouraged to conduct follow-up to collect treatment-related data and confirm the patient's survival status. Patients who have not been seen for at least 1 year will be considered lost to follow-up.
Sample Size	A total of 26 patients will be enrolled

Inclusion Criteria	Subjects must meet all the following inclusion criteria to be eligible for the study:<br>1. Male or female aged ≥18 years.<br>2. Confirmed diagnosis of HER2-positive urothelial carcinoma (HER2 immunohistochemistry (IHC) result: 2+ or 3+).<br>3. Meeting the criteria for very high-risk non-muscle invasive bladder cancer (VHR-NMIBC):<br>① Multiple/large high-grade T1 tumors (T1HG); ② Recurrent T1HG; ③ T1HG with carcinoma in situ (CIS); ④ Special histological variants (micropapillary urothelial carcinoma (UC), plasmacytoid UC, sarcomatoid UC, neuroendocrine variant of UC); ⑤ Prostatic urethral invasion; ⑥ BCG-unresponsive disease.<br>4. Meeting the criteria for extensive tumors: Multisite bladder mucosal biopsy showing >2 lesions and >3 pathological findings consistent with the above histopathological diagnoses; bladder tumors assessed as incompletely resectable by at least two senior clinical urologists (associate chief physician or above) by consensus; single or confluent lesions >5 cm in diameter, or multifocal lesions accounting for >30% of the total bladder surface area.<br>5. TUR-BT performed within 12 weeks prior to enrollment, with positive tumor cells detected by postoperative urine cytology.<br>6. Eastern Cooperative Oncology Group (ECOG) performance status score of 0-2.<br>7. Organ function meeting the following requirements (or with supportive care):<br>a. Hematological indices: Absolute neutrophil count ≥1.5×10^9/L, platelet count ≥100×10^9/L, hemoglobin ≥9.0 g/dL;<br>b. Liver function: Total bilirubin ≤1.5×upper limit of normal (ULN), alanine aminotransferase (ALT) and aspartate aminotransferase (AST) ≤2.5×ULN; for patients with intrahepatic metastasis, transaminases ≤5×ULN;<br>c. Renal function: Serum creatinine ≤1.5×ULN or creatinine clearance ≥50 mL/min (Cockcroft-Gault formula).<br>8. Voluntary participation in the study, signed informed consent

	form, and good compliance with follow-up.
Exclusion Criteria	1. Active, known or suspected autoimmune disease.<br>2. Known history of primary immunodeficiency.<br>3. Known history of allogeneic organ transplantation or allogeneic hematopoietic stem cell transplantation.<br>4. Pregnant or lactating female patients.<br>5. Untreated acute or chronic active hepatitis B or C infection. Patients receiving antiviral therapy may be eligible based on the investigator's judgment according to individual conditions with viral load monitoring.

	6. Use of immunosuppressive drugs within 4 weeks prior to the start of treatment, excluding nasal and inhaled corticosteroids or physiological doses of systemic corticosteroids (i.e., ≤10 mg/day prednisolone or equivalent physiological doses of other corticosteroids).<br>7. Known or suspected hypersensitivity to Tislelizumab or Disitamab Vedotin.<br>8. Definite history of active tuberculosis.<br>9. Participation in other clinical studies at the same time.<br>10. Sexually active males or females of childbearing potential not using reliable contraceptive measures.<br>11. Uncontrolled concurrent diseases, including but not limited to:<br>a. HIV infection (positive HIV antibody).<br>b. Severe infection in active stage or with poor clinical control.<br>c. Evidence of severe or uncontrolled systemic diseases (e.g., severe mental/neurological diseases, epilepsy or dementia, unstable or decompensated respiratory/cardiovascular/hepatic/renal diseases, uncontrolled hypertension [i.e., hypertension ≥CTCAE Grade 2 despite drug treatment]).<br>d. Active bleeding or newly diagnosed thrombotic disease receiving therapeutic doses of anticoagulants, or bleeding tendency (except those whose symptoms can be controlled with supportive care).
Statistical Considerations	SPSS 20.0 statistical analysis software will be used for all statistical analyses. All statistical tests are two-sided, and a P-value ≤0.05 is considered statistically significant. A 95% confidence interval (CI) will be used.<br>Continuous variables are described as mean ± standard deviation or median (minimum, maximum). Paired t-tests are used to compare intra-group differences before and after treatment relative to baseline values at screening. Categorical variables are described as frequency (constituent ratio). Chi-square test with exact probability method or nonparametric tests are used to analyze changes before and after treatment. Time-to-event variables are analyzed using the Kaplan-Meier method.

1 Study Background

1.1 Introduction

With the extension of human life expectancy and changes in lifestyle, malignant tumors have become a major disease seriously threatening human health and the leading cause of death worldwide. It is estimated that there were 18.1 million new cancer cases and 9.6 million cancer deaths worldwide in 2018. In China, bladder cancer ranked 13th in incidence in the 2015 cancer statistics, with an incidence rate of 8.83 per 100,000 in males, ranking 7th among male malignant tumors in China. Approximately 75% of bladder cancer (BC) cases are non-muscle invasive bladder cancer (NMIBC). The first major breakthrough in the clinical treatment of metastatic urothelial carcinoma (mUC) in the past 30 years: Bladder cancer (BC) is the 9th most common cancer type worldwide, with more than 430,000 new cases and over 165,000 deaths each year, and the incidence rate in males is three times that in females. Most bladder cancers are diagnosed at an early stage, but the disease has a very high rate of recurrence and progression, with approximately 78% of patients experiencing disease recurrence or progression within 5 years. According to histological classification, bladder cancer can be divided into urothelial carcinoma (UC), squamous cell carcinoma, adenocarcinoma, and less common metastatic carcinoma, small cell carcinoma, mixed carcinoma, carcinosarcoma, etc. Among them, UC is the most common type, accounting for about 90% of all bladder cancer cases. In 2009, the International Union Against Cancer classified bladder cancer into non-muscle invasive bladder cancer (NMIBC, Tis/Ta/T1 stage) and muscle invasive bladder cancer (MIBC, T2 stage and above) based on tumor stage. For patients with high-risk NMIBC, the current standard treatment is intravesical bacillus Calmette-Guerin (BCG) instillation after transurethral resection of bladder tumor (TUR-BT). However, a large proportion of patients receiving the above treatment still experience tumor recurrence or progression within the first 5 years, and radical cystectomy is the routinely recommended treatment, which significantly reduces the quality of life of patients.

Like melanoma and non-small cell lung cancer, urothelial carcinoma is a tumor with high somatic mutation frequency and high antigen expression, making it an optimal target for immune checkpoint inhibitors (ICIs). By binding to these targets, ICIs restore the immune response of T cells to tumor cells, invalidate the immune escape strategies adopted by tumors, and achieve therapeutic effects. ICIs have become the standard second-line treatment for advanced unresectable urothelial carcinoma.

With the extensive development of clinical studies on ICIs in the first-line treatment of advanced unresectable urothelial carcinoma, adjuvant treatment of high-risk patients after

radical cystectomy, and neoadjuvant treatment of MIBC, the efficacy and safety of comprehensive treatment regimens centered on ICIs have been widely recognized. However, there are still few clinical studies on the application of immune checkpoint inhibitors in patients with high-risk NMIBC (HR-NMIBC) at this stage. Among them, KEYNOTE-057, a Phase II study enrolling NMIBC patients with BCG-unresponsive disease, reported a CR rate of 41% and an incidence of Grade 3-4 treatment-related adverse events of only 13%.

However, the efficacy of ICI monotherapy is closely related to PD-L1 expression, and the positive expression rate of PD-L1 in bladder cancer patients is relatively low, only 15.1-23.5%. Therefore, ICI combination therapy regimens have become the main direction of clinical exploration and have achieved a higher tumor response rate, but drug-related adverse events have also increased significantly.

In addition, HER2 overexpression in bladder cancer is closely associated with tumor progression and poor prognosis, although HER2 genomic amplification is not a common mechanism. Previously, HER2-targeted monoclonal antibodies and tyrosine kinase inhibitors (TKIs) have not shown clinical benefits in mUC. At present, Disitamab Vedotin (RC48), an antibody-drug conjugate (ADC) independently developed in China, has been approved for marketing, and on January 5, 2022, it was approved by the National Medical Products Administration (NMPA) of China for the treatment of patients with locally advanced or metastatic UC with HER2 expression (IHC 2+ or 3+) who have received prior systemic chemotherapy. Antibody-drug conjugates are designed to selectively deliver potent cytotoxic drugs to antigen-expressing tumor cells by linking cytotoxins to monoclonal antibodies. RC48-ADC is a novel humanized anti-HER2 antibody conjugated to monomethyl auristatin E (MMAE) via a cleavable linker, which can selectively deliver the anticancer agent MMAE to HER2-positive tumor cells. Compared with other traditional chemotherapeutic drugs, it has fewer side effects while inhibiting or killing tumors, and its safety and efficacy in the treatment of patients with locally advanced or metastatic UC have been confirmed by several clinical studies. However, there are no relevant clinical studies reporting its safety and efficacy in patients with high-risk non-muscle invasive bladder cancer (HR-NMIBC).

In clinical practice, a large number of NMIBC patients have superficial bladder tumors characterized by multiple lesions, large area, and large volume, which cannot be completely resected by routine TUR-BT. Radical cystectomy is the only treatment option for these patients. Although the survival time of patients is prolonged, the quality of life of patients is significantly reduced after surgery. Relevant studies have found that HER2 overexpression

in bladder cancer often occurs in CIS and multifocal tumors and indicates a high risk of tumor recurrence. With the rapid progress of drug research and treatment concepts, whether the above patients can achieve long-term tumor control while preserving the bladder has become a hot spot in clinical research.

In response to the above problems, our research team launched a single-center, single-arm, investigator-initiated prospective clinical study (NCT04730232) in July 2020, using Tislelizumab combined with nab-paclitaxel for the treatment of HR-NMIBC unresectable by TUR-BT. As of January 2022, the interim analysis results of this study showed that the complete response rate of patients reached 56%, the bladder preservation rate was as high as 78.6%, and the incidence of Grade 3-4 drug-related adverse events was less than 2%. However, this single-center clinical study enrolled a small number of patients with a short follow-up time, and no biomarkers significantly associated with efficacy have been identified, which may have a large selection bias. In addition, it cannot provide high-level evidence-based medical evidence for the efficacy and safety of the Tislelizumab combined with low-dose nab-paclitaxel regimen in a short time.

1.2 Disease Background

1.2.1 Research Progress of RC48-ADC in Urothelial Carcinoma

In 2021, the results of an open-label, single-arm, multicenter, Phase II study (RC48-C009) evaluating the efficacy and safety of RC48-ADC monotherapy in patients with HER2-overexpressing unresectable, locally advanced or metastatic urothelial carcinoma who progressed during or after platinum-, gemcitabine-, and paclitaxel-based chemotherapy were presented at the ASCO GU Meeting. Patients received treatment until disease progression, intolerable toxicity, discontinuation, or study termination. The results showed that a total of 64 patients were enrolled from December 2018 to September 2020, with a median age of 62.5 years. Most patients (89.1%) had visceral metastases, 55 patients (85.9%) had received ≥2 lines of treatment, and 19 patients (29.7%) had received PD-1/PD-L1 inhibitors.

Efficacy analysis data showed that the confirmed objective response rate (cORR) of RC48-ADC in patients with HER2-overexpressing unresectable, locally advanced or metastatic urothelial carcinoma assessed by the Blinded Independent Review Committee (BIRC) was 50.0% (95% CI: 37.2, 62.8), disease control rate (DCR) was 76.6% (95% CI: 64.3, 86.2), median duration of response (DOR) was 8.3 months (95% CI: 4.2, 13.7), median PFS was 5.1 months (95% CI: 4.0, 6.9), 6-month PFS rate was 41.3% (95% CI: 28.3, 53.8), and median OS was 14.2 months (95% CI: 8.7, 19.2).

In this study, the most common adverse events of RC48 were AST elevation (46.9%),

decreased white blood cell count (45.3%), decreased neutrophil count (42.2%), and hypoesthesia (42.2%). The most common Grade 3/4 treatment-related adverse events (TRAEs) were neutropenia (10.9%), hypoesthesia (7.8%), and elevated γ-GGT (7.8%). There were 4 cases (6.3%) of serious adverse events (SAEs), including fever (1.1%), hypoesthesia (1.1%), hyperglycemia (1.1%), and anemia (1.1%).

This Phase II trial confirmed the efficacy of RC48 in patients with HER2-overexpressing UC, with a high response rate (cORR 50.0%) and a long median DOR of 8.3 months. The most common adverse events were AST elevation, decreased white blood cell count, decreased neutrophil count, and hypoesthesia. According to CTCAE, most of these adverse events were Grade 1-2, tolerable and manageable.

In 2022, the updated results of a two-arm trial (RC48-C014) of RC48 combined with Toripalimab in the treatment of locally advanced or metastatic urothelial carcinoma were presented at the ASCO GU Meeting. As of September 23, 2021, 32 patients had been enrolled in this study and received RC48 + Toripalimab treatment. The results showed that at the data cutoff, the investigator-assessed confirmed ORR of patients reached 75% (95% CI: 50.9, 91.3), of which 15% (95% CI: 75.1, 99.9) of patients achieved complete response (CR), and DCR was 95% (95% CI: 75.1, 99.9). Favorable efficacy was observed regardless of prior treatment lines, HER2 and PD-L1 expression status. In terms of safety, among the 32 patients receiving study treatment, the most common treatment-related adverse events were anorexia (72%), fatigue (56%, 8% ≥Grade 3), elevated transaminases (56%, 4% ≥Grade 3), peripheral sensory neuropathy (56%), alopecia (52%), nausea (36%), and anemia (32%). The most common immune-related AEs were pneumonitis (20%). RC48 combined with Toripalimab showed favorable efficacy and manageable safety, and further evaluation is ongoing. However, data on the safety and efficacy of RC48 combined with other immune checkpoint inhibitors are limited, and there are no relevant clinical studies reporting the safety and efficacy of RC48 in patients with high-risk non-muscle invasive bladder cancer (HR-NMIBC).

1.3 Study Drug: Disitamab Vedotin

1.3.1 Mechanism of Action of Disitamab Vedotin

Disitamab Vedotin is a novel HER2-targeted antibody-drug conjugate (ADC) composed of a recombinant humanized HER2 IgG1 monoclonal antibody conjugated to the microtubule inhibitor monomethyl auristatin E (MMAE) via a linker. After the antibody portion of Disitamab Vedotin binds to the extracellular domain of HER2 on the cell surface, the ADC complex is internalized into the cell and transported to the lysosome. The linker is

cleaved by enzymes to release the microtubule inhibitor MMAE, which disrupts the intracellular microtubule network, leading to cell cycle arrest in mitosis and cell apoptosis. In addition, in vitro studies have shown that Disitamab Vedotin can inhibit HER2 receptor signaling and exert antibody-dependent cell-mediated cytotoxicity (ADCC).

1.3.2 Clinical Study Results of Disitamab Vedotin

An open-label, multicenter, Phase II study (doi:10.1158/1078-0432.CCR-20-2488) evaluated the efficacy and safety of Disitamab Vedotin monotherapy in the treatment of patients with HER2-positive locally advanced or metastatic urothelial carcinoma. A total of 43 patients received Disitamab Vedotin at a dose of 2.0 mg/kg intravenously every 2 weeks until disease progression, intolerable toxicity, death, or withdrawal of consent. Enrolled patients must have at least one measurable lesion defined by the investigator according to RECIST Version 1.1. Patients were aged 18 to 80 years with histologically confirmed, unresectable, locally advanced or metastatic HER2-positive (IHC 3+ or 2+) urothelial carcinoma. Subjects must have received at least one line of systemic chemotherapy. In the event of treatment-related adverse events (TRAEs), doses were modified and interrupted (for up to 28 days) until these events resolved to Grade 0/1 or baseline. Toxicity was managed with supportive care. All patients were followed up every 12 weeks after the last treatment dose. Clinical responses were assessed by the Blinded Independent Review Committee (BIRC) according to RECIST Version 1.1 at baseline and every 6 weeks, regardless of dose delay or interruption, until documented disease progression or death. At baseline, most patients (83.7%) had confirmed visceral metastases, including 22 cases (51.2%) of lung metastases and 20 cases (46.5%) of liver metastases. Among the 43 patients, 41 patients had previously received platinum-based regimens (35 patients received cisplatin, 11 patients received carboplatin, 5 patients received both cisplatin and carboplatin), 8 patients received PD-1/PD-L1 blockade therapy after platinum chemotherapy failure (7 patients with cisplatin, 1 patient with carboplatin), and no patients received HER2-targeted therapy. Tumor responses assessed by BIRC are shown in the table below.。

Tumor Responses Assessed by BIRC

Endpoint	RC48-ADC (N=43)
Best Overall Response (BOR), n (% ; 95% CI)	22 (51.2%; 35.5%–66.7%)
Complete Response (CR)	0
Partial Response (PR)	22 (51.2%; 35.5%–66.7%)
Stable Disease (SD)	17 (39.5%; 25.0%–55.6%)
Progressive Disease (PD)	3 (7.0%; 1.5%–19.1%)

Not Evaluable	1 (2.3%; 0.1%–12.3%)
Confirmed Objective Response Rate (cORR), (95% CI)	51.2% (35.5%–66.7%)
HER2 IHC 3+ or IHC 2+ and FISH positive	60.0% (36.1%–80.9%)*
HER2 IHC 2+ and FISH negative	40.0% (19.1%–63.9%)*
Duration of Response (DoR), months (95% CI)	6.9 (4.7–10.8)

*The ORR difference between the two groups was 20%, P=0.21 (>0.05).

1.4 Research Progress of RC48-ADC in the Treatment of HER2-Positive Urothelial Carcinoma

In 2021, the results of an open-label, single-arm, multicenter, Phase II study (RC48-C009) evaluating the efficacy and safety of RC48-ADC monotherapy in patients with HER2-overexpressing unresectable, locally advanced or metastatic urothelial carcinoma who progressed during or after platinum-, gemcitabine-, and paclitaxel-based chemotherapy were presented at the ASCO GU Meeting. The results showed a high objective response rate of 50.0%, a disease control rate of 76.6%, a mean duration of response of 8.3 months, a median progression-free survival of 5.1 months, a 6-month progression-free survival rate of 41.3%, and a median overall survival of 14.2 months.

In 2022, the updated results of a two-arm trial (RC48-C014) of RC48 combined with Toripalimab in the treatment of locally advanced or metastatic urothelial carcinoma were presented at the ASCO GU Meeting, showing a good synergistic effect of RC48 + Toripalimab. As of September 23, 2021, the investigator-assessed confirmed ORR of patients was 75%, of which 15% of patients achieved CR, and DCR was 95%, which is the best efficacy for mUC to date. Favorable efficacy was observed regardless of prior treatment lines, HER2 and PD-L1 expression status. In terms of safety, the vast majority of treatment-related adverse events in patients were Grade 1-2. RC48 combined with Toripalimab showed favorable efficacy and manageable safety, and further evaluation is ongoing.

2 Study Objectives

2.1 Primary Study Endpoint

- Evaluate the Complete Response Rate of High-Risk Disease (HR-CRR) of

Disitamab Vedotin combined with Tislelizumab in the treatment of HER2-positive VHR-NMIBC unresectable by TUR-BT.

2.2 Secondary Study Endpoints

- Evaluate the Complete Response Rate of Any Disease (CRR) of Disitamab Vedotin combined with Tislelizumab in the treatment of HER2-positive VHR-NMIBC unresectable by TUR-BT.
- Evaluate the Duration of Complete Response (DOR) of Any Disease and High-Risk Disease of Disitamab Vedotin combined with Tislelizumab in the treatment of HER2-positive VHR-NMIBC unresectable by TUR-BT.
- Evaluate the Progression-Free Survival (PFS) and Overall Survival (OS) of Disitamab Vedotin combined with Tislelizumab in the treatment of HER2-positive VHR-NMIBC unresectable by TUR-BT.
- Evaluate the safety, incidence of Serious Adverse Events (SAEs), and incidence of Treatment-Related Adverse Events (TRAEs) of Disitamab Vedotin combined with Tislelizumab (Adverse events are graded according to the Common Terminology Criteria for Adverse Events (CTCAE) Version 5.0).

3 Overall Study Design

This is an interventional clinical study designed to evaluate the efficacy and safety of Disitamab Vedotin combined with Tislelizumab in the treatment of HER2-positive very high-risk non-muscle invasive bladder cancer (VHR-NMIBC) unresectable by transurethral resection of bladder tumor (TUR-BT).

Adverse events will be monitored during the study and graded according to the National Cancer Institute (NCI) Common Terminology Criteria for Adverse Events (CTCAE) Version 5.0.

All eligible patients meeting the inclusion and exclusion criteria will be invited to participate in the study. Patients will be considered in the study unless they die, withdraw from the study, are lost to follow-up, or the study is terminated. Patients are not required to make special visits or undergo evaluations for the study, and evaluations will be conducted by investigators based on clinical practice.

As an observational study, the diagnosis and treatment procedures listed in the protocol are clinical recommended procedures and not mandatory. The treatment and clinical visit schedule of enrolled patients will be determined by clinicians based on

routine practice. Subject information, including efficacy and safety data, will be collected in accordance with routine diagnosis and treatment procedures. Although the protocol does not mandate clinical visits, for patients who have not been seen for at least 6 months, investigators are encouraged to conduct follow-up to collect treatment-related data and confirm the patient's survival status. Patients who have not been seen for at least 1 year will be considered lost to follow-up.

4 Study Population

4.1 Inclusion Criteria

Subjects must meet all the following inclusion criteria to be eligible for the study:

1. Male or female aged ≥18 years.
2. Confirmed diagnosis of HER2-positive urothelial carcinoma (HER2 immunohistochemistry (IHC) result: 2+ or 3+).
3. Meeting the criteria for very high-risk non-muscle invasive bladder cancer (VHR-NMIBC):

   ① Multiple/large high-grade T1 tumors (T1HG); ② Recurrent T1HG; ③ T1HG with carcinoma in situ (CIS); ④ Special histological variants (micropapillary urothelial carcinoma (UC), plasmacytoid UC, sarcomatoid UC, neuroendocrine variant of UC); ⑤ Prostatic urethral invasion; ⑥ BCG-unresponsive disease.
4. Meeting the criteria for extensive tumors: Multisite bladder mucosal biopsy showing >2 lesions and >3 pathological findings consistent with the above histopathological diagnoses; bladder tumors assessed as incompletely resectable by at least two senior clinical urologists (associate chief physician or above) by consensus; single or confluent lesions >5 cm in diameter, or multifocal lesions accounting for >30% of the total bladder surface area.
5. Willing to provide blood, urine and tissue samples for testing (including PD-L1 expression, immunohistochemistry, tumor mutational burden, DNA and RNA detection, etc.).
6. Eastern Cooperative Oncology Group (ECOG) performance status score of 0-2.
7. Organ function meeting the following requirements (or with supportive care):

   a. Hematological indices: Absolute neutrophil count $\geq 1.5\times 10^9$/L, platelet count $\geq 100\times 10^9$/L, hemoglobin ≥9.0 g/dL;

   b. Liver function: Total bilirubin ≤1.5×upper limit of normal (ULN), alanine aminotransferase (ALT) and aspartate aminotransferase (AST) ≤2.5×ULN; for patients with intrahepatic metastasis, transaminases ≤5×ULN;

c. Renal function: Serum creatinine ≤1.5×ULN or creatinine clearance ≥50 mL/min (Cockcroft-Gault formula).

8. Voluntary participation in the study, signed informed consent form, and good compliance with follow-up.

4.2 Exclusion Criteria

1. Active, known or suspected autoimmune disease.
2. Known history of primary immunodeficiency.
3. Known history of allogeneic organ transplantation or allogeneic hematopoietic stem cell transplantation.
4. Pregnant or lactating female patients.
5. Untreated acute or chronic active hepatitis B or C infection. Patients receiving antiviral therapy may be eligible based on the investigator's judgment according to individual conditions with viral load monitoring.
6. Use of immunosuppressive drugs within 4 weeks prior to the start of treatment, excluding nasal and inhaled corticosteroids or physiological doses of systemic corticosteroids (i.e., ≤10 mg/day prednisolone or equivalent physiological doses of other corticosteroids).
7. Known or suspected hypersensitivity to Tislelizumab or Disitamab Vedotin.
8. Definite history of active tuberculosis.
9. Participation in other clinical studies at the same time.
10. Sexually active males or females of childbearing potential not using reliable contraceptive measures.
11. Uncontrolled concurrent diseases, including but not limited to:

a) HIV infection (positive HIV antibody).

b) Severe infection in active stage or with poor clinical control.

c) Evidence of severe or uncontrolled systemic diseases (e.g., severe mental/neurological diseases, epilepsy or dementia, unstable or decompensated respiratory/cardiovascular/hepatic/renal diseases, uncontrolled hypertension [i.e.,

hypertension ≥CTCAE Grade 2 despite drug treatment]).

d) Active bleeding or newly diagnosed thrombotic disease receiving therapeutic doses of anticoagulants, or bleeding tendency (except those whose symptoms can be controlled with supportive care)

4.3 Criteria for Patient Withdrawal from the Study

Investigators have the right to request patients to withdraw from the study at any time. In addition, patients have the right to withdraw from the study at any time for any reason. Reasons for withdrawal from the study may include, but are not limited to:

a) Patient withdrawal of consent

b) Investigator judges that termination of treatment is in the best interest of the patient

c) Patient lost to follow-up

Every effort will be made to obtain information about patients who withdraw from the study. For patients lost to follow-up, attempts will be made to determine their final status (i.e., withdrawal of consent or death). The primary reason for withdrawal from the study should be documented in the corresponding Case Report Form (CRF). However, no follow-up will be conducted on patients for any reason after withdrawal of consent.

5 Study Drugs and Treatment Regimen

5.1 Treatment Regimen

5.1.1 Disitamab Vedotin Treatment Regimen

The total duration of treatment in this study is 1 year, with a treatment cycle of 21 days. The specific treatment methods and drug dosages are shown in the table below.

Treatment Regimen of Disitamab Vedotin Combined with Tislelizumab

Drug Name	Dose	Administration Method	Treatment Course/Cycle
Disitamab Vedotin	120mg	Intravenous infusion	21 days per cycle, administered on Day 1; maximum of 8 cycles of treatment
Tislelizumab	200mg	Intravenous infusion	21 days per cycle, administered on Day 2; maximum treatment duration of 1 year

5.1.2 Administration of Disitamab Vedotin

Ingredients

Active ingredient: Disitamab Vedotin is an antibody-drug conjugate consisting of three parts: (1) Anti-human epidermal growth factor receptor 2 extracellular domain (HER2 ECD) antibody (2) Linker (MC-Val-Cit-PAB) (3) Cytotoxin monomethyl auristatin E (MMAE)

Excipients: L-Histidine hydrochloride, Mannitol, Sucrose, Polysorbate 80, Sodium hydroxide.

The product is a white to pale yellow loose powder, and a colorless to pale yellow clear liquid after reconstitution.

Specifications

Each vial contains 60 mg of Disitamab Vedotin.

After reconstitution, each milliliter of the solution contains 10 mg of Disitamab Vedotin.

Lyophilized preparation.

Drug Preparation

The product should be reconstituted, diluted and administered at room temperature within 4 hours after removal. The specific steps are as follows:

Reconstitution: The product is a lyophilized preparation. Reconstitute each vial (60 mg) with 6 mL of sterile water for injection to a concentration of 10 mg per mL of Disitamab Vedotin. Add the sterile water for injection slowly along the side of the vial to minimize foam formation. During reconstitution, place the vial at room temperature and rotate gently for about 60 seconds; vigorous shaking is strictly prohibited. Allow to stand until the foam subsides. After the drug is dissolved, rotate gently again to mix the solution thoroughly. Reconstitution is usually completed within 10 minutes after adding sterile water for injection. The reconstituted solution is a colorless to pale yellow clear liquid.

Discard if visible particles are observed.

Since the product contains no preservatives, discard any unused drug remaining in the vial.

Dilution: Add the reconstituted solution obtained in the above steps to 0.9% sodium chloride injection or 5% glucose injection for dilution according to the drug dose calculated based on body weight.

The method for calculating the administered dose based on body weight is as follows:

If the required drug dose calculated based on body weight is a multiple of the whole vial, aspirate all the reconstituted solution from the vial with a syringe;

If the required drug dose calculated based on body weight is not a multiple of the whole vial, it is recommended to round the volume of the non-integer vial to 0.1 mL for calculation, and aspirate the required volume with a syringe。

Administration Regimen:

Administered by intravenous infusion; intravenous bolus injection or rapid intravenous injection is prohibited. The infusion duration is 30-90 minutes (60 minutes is usually recommended). If an infusion-related reaction or hypersensitivity reaction occurs during infusion, slow down or interrupt the infusion and/or provide appropriate medical treatment. Discontinue the drug immediately for life-threatening infusion-related reactions.

Storage

Store and transport at 2-8°C in the dark.

Packaging

1 vial/box

Packaged in neutral borosilicate glass injection vials with bromobutyl rubber stoppers, each vial contains 60 mg of Disitamab Vedotin.

Validity Period

24 months

5.1.3 Administration of Tislelizumab

The main active ingredient of Tislelizumab is recombinant fully human anti-programmed death receptor 1 monoclonal antibody at a concentration of 10 mg/mL. The product is a clear to slightly opalescent, colorless to pale yellow liquid. Excipients include disodium citrate dihydrate, citric acid monohydrate, L-histidine hydrochloride monohydrate, L-histidine, trehalose dihydrate, polysorbate 20 and water for injection. Tislelizumab finished product is stored at 2~8°C in the dark with a validity period of 18 months.

For intravenous infusion of Tislelizumab, the first infusion duration should be no less than 60 minutes; if well tolerated, the subsequent infusion duration should be no less than 30 minutes. Administration by intravenous bolus injection or single rapid intravenous injection is not allowed. The dilution instructions for the drug before administration are as follows (see the package insert for details):

Solution Preparation and Infusion

• Do not shake the vial.

• Allow the vial to reach room temperature (25°C or below) before use.

• After removal from the refrigerator, the vial can be left at room temperature (25°C or below) for a maximum of 2 hours before dilution.

• Visually inspect the injection for suspended particles and discoloration before administration. The product is a clear to slightly opalescent, colorless to pale yellow liquid. Discard the drug if visible particles or abnormal color are observed.

• Aspirate 2 vials of the product injection (200 mg in total) and transfer to an intravenous infusion bag containing 9 mg/mL (0.9%) sodium chloride solution to prepare a final concentration ranging from 1~5 mg/mL. Gently invert the diluent to mix well.

• The product contains no preservatives. It is recommended to prepare the solution immediately after removal from the refrigerator. If immediate use is not possible, the diluted solution can be stored for no more than 24 hours, including storage at refrigerated conditions (2～8°C) for no more than 20 hours and recovery to room temperature (25°C and below) with completion of infusion within 4 hours.

• Do not freeze the product.

• Do not administer other drugs through the same infusion line at the same time.

The product is for single use only. Discard any unused drug remaining in the vial.

5.2 Dose Adjustment

5.2.1 Dose Adjustment of Tislelizumab

Dose Adjustment Regimen for Tislelizumab

Tislelizumab-Related Adverse Event	Severity	Dose Adjustment
Pneumonitis	Grade 2	Withhold dosing[a]
	Grade 3/4 or recurrent Grade 2	Permanently
Diarrhea/Colitis	Grade 2/3	Withhold dosing[a]
	Grade 4	Permanently
Hepatitis	For subjects with normal baseline ALT/AST or TBIL: Grade 2 elevation of AST/ALT (3-5×ULN) or TBIL (1.5-3×ULN); For subjects with baseline AST/ALT or TBIL >ULN: ≥50% elevation of AST/ALT or TBIL (meeting Grade 2 requirements) with duration ≤7 days	Withhold dosing[a]

	For subjects with normal baseline ALT/AST or TBIL: Grade 3/4 elevation of AST/ALT (>5×ULN) or TBIL (>3×ULN); For subjects with baseline AST/ALT or TBIL >ULN: ≥50% elevation of AST/ALT or TBIL (meeting Grade 3/4 requirements) with duration ≥7 days	Permanently discontinue
Endocrine disease	Symptomatic Grade 2/3 hypothyroidism<br>Grade 2/3 hyperthyroidism<br>Grade 2/3 hypophysitis<br>Grade 2 adrenal insufficiency<br>Grade 3 hyperglycemia or Type 1 diabetesWithhold dosing[b]Grade 4 hypothyroidism	Withhold dosing[b]
	Grade 4 hypothyroidism	Permanently
Cutaneous adverse reaction	Grade 3	Withhold dosing[a]
	Grade 4, Stevens-Johnson syndrome (SJS) or toxic epidermal necrolysis (TEN)	Permanently discontinue
Thrombocytopenia	Grade 3	Withhold dosing[a]
	Grade 4	Permanently discontinue

Other immune-related adverse events	Grade 3/4 elevated serum amylase or lipase<br>Grade 2/3 pancreatitis<br>Grade 2 myocarditis[c]<br>Grade 2/3 first-occurrence other immune-related adverse events	Withhold dosing[a]
	Grade 4 pancreatitis or recurrent pancreatitis of any grade<br>Grade 3/4 myocarditis<br>Grade 3/4 encephalitis<br>Grade 4 first-occurrence other immune-related adverse events[d]	Permanently discontinue
Recurrent or persistent adverse events	Recurrent Grade 3/4 (excluding endocrine diseases)<br>Grade 2/3 adverse events within 12 weeks after the last dose<br>Failure to improve to Grade 0~1 (excluding endocrine diseases)<br>Corticosteroids not reduced to ≤10 mg/day prednisolone equivalent within 12 weeks after the	Permanently discontinue

[a]: Resume dosing after symptoms improve to Grade 0-1 or baseline.

*The safety of resuming Tislelizumab treatment after myocarditis improves to Grade 0-1 is unclear.

[b]: Hypophysitis, adrenal insufficiency, hypothyroidism/hyperthyroidism and Type 1 diabetes can be resumed if well-controlled with only physiological hormone replacement therapy.

[c]: The safety of resuming Tislelizumab treatment after myocarditis improves to Grade 0~1 is unclear.

[d]: For Grade 4 abnormal laboratory test results, the decision to discontinue treatment should be based on accompanying clinical symptoms/signs and the investigator's clinical judgment.

5.2.2 Dose Adjustment of Disitamab Vedotin

Dose and cycle adjustments of Disitamab Vedotin and Tislelizumab are not recommended during the study. For suspension and permanent discontinuation of Disitamab Vedotin, dose adjustment shall be performed in accordance with the drug label.

5.2.3 Management of Infusion Reactions Related to Disitamab Vedotin and Tislelizumab

Disitamab Vedotin and Tislelizumab may cause severe or life-threatening infusion reactions, including severe hypersensitivity or anaphylaxis. Signs and symptoms usually occur during or shortly after drug infusion and usually resolve completely within 24 hours after completion of infusion. The management guidelines for infusion reactions related to Disitamab Vedotin and Tislelizumab are shown in the table below.

Management Guidelines for Infusion Reactions Related to Disitamab Vedotin and Tislelizumab

**NCI CTCAE Grade**	Treatment	Premedication for Subsequent Dosing
**Grade 1**<br>Mild reaction; no infusion interruption required; no intervention required	Strengthen vital sign monitoring according to the patient's medical indications until the investigator deems the subject's condition stable.	None

**Grade 2**<br><br>Treatment or infusion interruption required, but rapid response to symptomatic treatment (e.g., antihistamines, non-steroidal anti-inflammatory drugs [NSAIDs], anesthetics, intravenous fluid replacement) is achieved; prophylactic medication for ≤24 hours is required	Stop infusion and monitor symptoms.<br><br>Other appropriate drug treatments may include, but are not limited to:<br><br>• Intravenous fluid replacement<br><br>• Antihistamines<br><br>• NSAIDs<br><br>• Acetaminophen, anesthetics<br><br>Strengthen vital sign monitoring according to the patient's medical indications until the investigator deems the subject's condition stable.<br><br>If symptoms resolve within one hour after stopping drug infusion, the infusion can be restarted at 50% of the original infusion rate (e.g., reduced from 100 mL/h to 50 mL/h). Otherwise, suspend dosing until symptoms resolve, and the subject should receive premedication before the next scheduled dosing.<br><br>Subjects who experience Grade 2 toxicity despite adequate premedication should permanently discontinue further study drug treatment.	Subjects may receive the following premedication 1.5 hours (±30 minutes) before Disitamab Vedotin infusion:<br><br>• Oral 50 mg diphenhydramine (or equivalent dose of antihistamine).<br><br>• Oral 500-1000 mg acetaminophen (or equivalent dose of antipyretic).

**Grade 3/4**<br>**Grade 3:**<br>Long duration (i.e., no rapid response after symptomatic medication and/or short-term infusion interruption); symptom recurrence after initial improvement; hospitalization required due to other clinical sequelae (e.g., renal impairment, pulmonary infiltration)<br>**Grade 4:**<br>Life-threatening; requiring vasopressors or ventilatory support	Stop infusion.<br>Other appropriate drug treatments may include, but are not limited to:<br>• Epinephrine**<br>• Intravenous fluid replacement<br>• Antihistamines<br>• NSAIDs<br>• Acetaminophen, anesthetics<br>• Oxygen<br>• Vasopressors<br>• Corticosteroids<br>Strengthen vital sign monitoring according to the patient's medical indications until the investigator deems the subject's condition stable.<br>Hospitalization may be required.<br>**Administer epinephrine immediately if anaphylaxis occurs.<br>Subjects should permanently discontinue further study drug treatment.	No subsequent dosing

5.3 Toxicity Management Principles

5.3.1 Toxicity Management Principles for RC48-ADC

Disitamab Vedotin is a novel HER2-targeted antibody-drug conjugate (ADC) composed of a recombinant humanized HER2 IgG1 monoclonal antibody conjugated to the

microtubule inhibitor monomethyl auristatin E (MMAE) via a linker. After the antibody portion of Disitamab Vedotin binds to the extracellular domain of HER2 on the cell surface, the ADC complex is internalized into cells and transported to lysosomes. The linker is enzymatically cleaved to release the microtubule inhibitor MMAE, which disrupts the intracellular microtubule network, leading to mitotic cell cycle arrest and apoptosis. In addition, in vitro studies have shown that Disitamab Vedotin can inhibit HER2 receptor signaling and exert antibody-dependent cell-mediated cytotoxicity (ADCC). These treatment-related adverse events (TRAEs) may occur shortly after the first administration or months after the last administration of Disitamab Vedotin, and may simultaneously affect more than one body system, such as anorexia, fatigue, elevated ALT/AST, peripheral sensory neuropathy, neutropenia, intestinal obstruction, drug-induced liver injury, constipation, etc. Therefore, early detection and initiation of treatment are crucial to reducing complications. Based on existing clinical trial data, most TRAEs are reversible and can be managed by interrupting Disitamab Vedotin administration, administering glucocorticoids, and/or other supportive therapies. Once the above-mentioned AEs occur in subjects of this study, subjects' signs and symptoms shall be monitored, and relevant examinations such as endoscopy or skin biopsy shall be performed to identify the etiology. If no alternative etiology (such as disease progression, concurrent medications, and infections) is found and glucocorticoid and/or other immunosuppressive therapies are required, the above-mentioned AEs shall be considered related to systemic dysfunction caused by Disitamab Vedotin and diagnosed as TRAEs. Based on the severity of TRAEs, Disitamab Vedotin treatment shall be suspended or permanently discontinued, and glucocorticoids or other supportive therapies shall be administered.

5.3.2 Toxicity Management Principles for Immune Checkpoint Inhibitors

AEs associated with Tislelizumab exposure may have an immunological etiology. This is because the mechanism of action of Tislelizumab is to block the interaction between PD-1 and PD-L1, restore T cell activity, which may lead to autoimmune hyperactivity, thereby causing immune-related adverse events (irAEs). These irAEs may occur shortly after the first administration or months after the last administration of Tislelizumab, and may simultaneously affect more than one body system, such as immune-related pneumonitis, diarrhea/colitis, renal insufficiency, rash, hepatitis, endocrine diseases, and peripheral or central neuritis and other autoimmune AEs. Therefore, early detection and initiation of treatment are crucial to reducing complications. Based on existing clinical trial data, most irAEs are reversible and can be managed by interrupting Tislelizumab administration, administering glucocorticoids, and/or other supportive therapies. Once the above-mentioned

AEs occur in subjects of this study, subjects' signs and symptoms shall be monitored, and relevant examinations such as bronchoscopy, endoscopy, or skin biopsy shall be performed to identify the etiology. If no alternative etiology (such as disease progression, concurrent medications, and infections) is found and glucocorticoid and/or other immunosuppressive therapies are required (excluding endocrine events such as hyperthyroidism/hypothyroidism, hypophysitis, Type 1 diabetes, and adrenal insufficiency, which may not require immunosuppressive therapy but are still considered related to autoimmune hyperactivity caused by Tislelizumab), the above-mentioned AEs shall be considered related to immune system hyperactivity caused by Tislelizumab and diagnosed as irAEs. Based on the severity of irAEs, Tislelizumab treatment shall be suspended or permanently discontinued, and glucocorticoids shall be administered.

5.4 Prohibited Concomitant Therapies

a) For prohibited concomitant therapies related to Disitamab Vedotin, refer to the respective product instructions.
b) Live vaccine vaccination is prohibited within 30 days prior to the first administration of Tislelizumab and during the treatment period.

6 Study Evaluation and Procedures

6.1 Screening Period

a) Sign the Informed Consent Form (ICF)
b) Verify inclusion/exclusion criteria
c) Record demographic data, medical history, and prior medications
d) Record vital signs, height, and weight
e) Tumor molecular characteristics
f) Physical examination
g) Eastern Cooperative Oncology Group (ECOG) Performance Status (PS) score
h) 12-lead electrocardiogram (ECG)
i) Routine blood test, blood biochemistry, urinalysis
j) Myocardial enzymes
k) Coagulation function
l) Glycated hemoglobin (HbA1c)
m) Lipid profile

n) Adrenal cortical function

o) Thyroid function

p) HIV, HBV, and HCV testing

q) Fecal 16S rDNA sequencing

r) Cystoscopy

s) Tumor urinalysis (NMP22, urinary cytology; urine FISH, MRD testing, and other urine-based liquid biopsy techniques may be performed at the discretion of the investigator)

t) Tumor pathological examination (TUR-BT pathology)

u) Imaging examinations (PET-MR/PET-CT/MR/CT)

6.2 On-Treatment Visits

a) Record vital signs and weight

b) Physical examination

c) ECOG PS score

d) 12-lead ECG

e) Routine blood test, blood biochemistry, urinalysis

f) Myocardial enzymes

g) Coagulation function

h) Thyroid function

i) Fecal 16S rDNA sequencing

j) Adverse event assessment

k) Record concurrent medications

l) Tumor urinalysis (NMP22, urinary cytology; urine FISH, MRD testing, and other urine-based liquid biopsy techniques may be performed at the discretion of the investigator)

m) Imaging examinations (PET-MR/PET-CT/MR/CT)

6.3 Post-Treatment Evaluation

a) Record vital signs and weight

b) Physical examination

c) ECOG PS score

d) 12-lead ECG
e) Routine blood test, blood biochemistry, urinalysis
f) Myocardial enzymes
g) Coagulation function
h) Glycated hemoglobin (HbA1c)
i) Lipid profile
j) Adrenal cortical function
k) Thyroid function
l) Fecal 16S rDNA sequencing
m) Adverse event assessment
n) Record concurrent medications
o) Tumor urinalysis (NMP22, urinary cytology; urine FISH, MRD testing, and other urine-based liquid biopsy techniques may be performed at the discretion of the investigator)
p) Cystoscopy
q) Tumor pathological examination (see Section 6.4 for specific tissue acquisition methods)
r) Imaging examinations (PET-MR/PET-CT/MR/CT)

6.4 Methods for Obtaining Tissues for Tumor Pathological Examination

After 3 cycles of treatment, patients will undergo clinical examinations including cystoscopy. Based on the comprehensive assessment by the investigator, a decision will be made whether to perform surgical treatment. Surgical treatment includes but is not limited to radical cystectomy and transurethral resection of bladder tumor (TUR-BT).

6.5 Follow-Up After the Completion of Investigational Treatment

The time points for postoperative follow-up shall be based on clinical diagnosis and treatment practice.

Follow-up content includes:

a) Whether surgery was performed, surgical time, and surgical type
b) Vital signs, ECOG score, and physical examination
c) Tumor urinalysis (NMP22, urinary cytology; urine FISH, MRD testing may be performed at the discretion of the investigator)
d) Cystoscopy (including selective tissue biopsy) (performed every 3 months during follow-up and when clinically indicated)
e) Imaging examinations (PET-MR/PET-CT/MR/CT)
f) Concurrent medications and adverse events

g) New anti-tumor treatments

h) Survival status: For patients who have not been followed up for at least 6 months, telephone follow-up will be conducted to confirm the patient's survival status and collect relevant treatment data.

7 Efficacy Evaluation

7.1 Initial Efficacy Evaluation

A comprehensive assessment will be performed to evaluate efficacy approximately 3 months after the initiation of treatment. This comprehensive assessment includes transurethral resection biopsy, urine cytology, and imaging. The transurethral resection biopsy encompasses directed biopsies at the original tumor sites and any identified anomalies, along with random multi-point bladder biopsies. Male patients may undergo a biopsy of the prostatic urethra if necessary.

7.2 Follow-up

During the subsequent follow-up period, cystoscopy and urine cytology will be conducted every 3 months for the first 2 years, followed by assessments every 6 months for the next 3 years. Directed biopsies will be carried out in the event of abnormal cystoscopy findings, and random biopsies will be carried out in the event of abnormal urine cytology. Chest and pelvic imaging will be performed every 6 months for the first 2 years, followed by annual assessments for the next 3 years. Safety and toxicity profile will be assessed according to treatment-related adverse events (trAEs) using the National Cancer Institute Common Terminology Criteria for Adverse Events (CTCAE) version 5.0.

7.3 Safety Evaluation

Adverse events will be graded and recorded according to the NCI CTCAE (Version 5.0) during the study and follow-up period. Toxicity characteristics will be determined based on the following aspects: severity, causal relationship, toxicity grade, and measures taken for the investigational treatment, and a judgment will be made as to whether it is a treatment-related adverse event (TRAE).

8 Safety Reporting and Adverse Event Management

8.1 Definition of Adverse Event (AE)

An Adverse Event (AE) is defined as any unfavorable medical event that occurs in a subject after receiving a certain drug treatment in a clinical study, regardless of whether it is causally related to the investigational drug. AEs include but are not limited to the following situations:

Aggravation of existing medical conditions/diseases (including aggravation of

symptoms, signs, and laboratory abnormalities) prior to entry into the clinical study;

Any newly occurring unfavorable medical conditions (including symptoms, signs, and newly diagnosed diseases);

Abnormal laboratory test values or results with clinical significance.

8.2 Definition of Serious Adverse Event (SAE)

A Serious Adverse Event (SAE) refers to an adverse event that meets at least one of the following criteria:

Resulting in death, excluding death caused by the progression of the study indication disease.

Life-threatening (the "life-threatening" in the definition refers to an AE that poses a risk of death to the subject when it occurs, rather than an AE that may cause death if the event worsens).

Requiring hospitalization or prolongation of hospitalization, excluding the following situations:

√ Rehabilitation institutions

√ Nursing homes

√ Routine emergency department admission

√ Same-day surgery (e.g., outpatient/same-day/ambulatory surgery)

√ Hospitalization or prolongation of hospitalization unrelated to the deterioration of AE is not an SAE per se. For example: Admission due to an existing disease without new adverse events or aggravation of the existing disease (e.g., to check for persistent laboratory abnormalities since before the study); Hospitalization for administrative reasons (e.g., annual routine physical examination); Hospitalization required by the study protocol during the clinical trial (e.g., performing operations as required by the study protocol); Elective hospitalization unrelated to the deterioration of adverse events (e.g., elective surgery); Scheduled treatment or surgical procedures shall be documented in the entire study protocol and/or the subject's individual baseline data; Admission solely for blood product use.

Resulting in permanent or severe disability/incapacity.

Resulting in congenital anomalies/birth defects.

Other important medical events: defined as events that endanger the subject or require medical intervention to prevent any of the above situations.

8.3 Evaluation of Adverse Events

Investigators will evaluate the severity of all adverse events in accordance with the NCI Common Terminology Criteria for Adverse Events (CTCAE) Version 5.0. All adverse events, regardless of CTCAE grade, must be evaluated for whether they are serious adverse

events. For adverse events not specifically listed in NCI CTCAE, their severity can be evaluated with reference to the following table:。

Grade	Severity
1	Mild; asymptomatic or mild symptoms; clinical or diagnostic observation only; or no intervention required
2	Moderate; minimal, local, or non-invasive intervention required; or impairment of age-related instrumental activities of daily living
3	Severe or of significant clinical significance, but not immediately life-threatening; requiring hospitalization or prolongation of hospitalization; disabling; or impairment of activities of daily living
4	Life-threatening consequences or requiring emergency intervention
5	Death related to the adverse event

8.4 Documentation of Adverse Events

Investigators shall document AEs or SAEs using medical terms/concepts. Colloquialisms and abbreviations should be avoided. All AEs (including SAEs) shall be recorded on the adverse event form of the Case Report Form (CRF).

8.4.1 Time Window for AE Collection

Investigators will collect adverse event information at each follow-up visit. In accordance with the requirements stated in the study protocol, all adverse events, whether reported by patients or identified by researchers, shall be recorded in the patient's medical records and CRF.

All adverse events, including serious adverse events, whether observed by investigators or spontaneously reported by subjects, will be collected from enrollment until 30 days after the last administration. After 30 days of the last administration, investigators will only record serious adverse events considered related to the investigational drug.

8.4.2 Follow-Up of Adverse Events

Adverse events should be followed up until they return to baseline or Grade 0~1, or until the investigator deems it unnecessary to continue follow-up for reasonable reasons (e.g., irrecoverable or improved). If the adverse event cannot be recovered, a reasonable

explanation shall be recorded in the CRF. Regardless of whether it is related to the investigational drug, the recovery status and date of the subject's AE or SAE shall be recorded in the CRF and medical records.

8.4.3 Content of AE Documentation

Investigators shall completely document any adverse event, including diagnosis (if no diagnosis is available, record symptoms, signs including laboratory abnormalities), start and end dates and times (if applicable), CTCAE severity grade and changes (for Grade 3 or above events), whether it is a serious adverse event, measures taken for the investigational drug, treatment given for the AE, and the outcome of the event, as well as the relationship between the adverse event and the investigational drug.

8.5 Reporting of Serious Adverse Events (SAEs)

The reporting period for SAEs covers serious adverse events occurring from the signing of the informed consent to 30 days (inclusive) after the last administration. If an SAE occurs, whether it is an initial report or a follow-up report, the investigator must immediately complete the "Serious Adverse Event Report Form" and report it in accordance with Chinese regulatory requirements.

9 Statistical Considerations

9.1 Statistical Analysis Populations and Sample Size Estimation

Efficacy and safety analyses will be based on all enrolled patients who have received at least one cycle of Disitamab Vedotin or Disitamab Vedotin combined with Tislelizumab treatment.

Based on previous studies, the null hypothesis is that the complete response rate is 41%, and the alternative hypothesis is 70%; with α set at 0.05 and power at 80%, Simon's two-stage design is used for sample size calculation. The first stage requires enrolling 7 patients. If 4 patients achieve the complete response rate of high-risk disease, an additional 16 patients will be enrolled in the second stage. Considering patient dropout, a total of 26 patients need to be enrolled.

9.2 Statistical Analysis Methods

9.2.1 General Statistical Analysis Methods

Continuous variables are described as mean ± standard deviation or median (minimum, maximum). Categorical variables are described as frequency (constituent ratio). There is no formal hypothesis testing; 95% confidence interval (95% CI) will be calculated when necessary.

9.2.2 Baseline Characteristic Analysis

Demographic data, clinical baseline characteristics, and molecular characteristics of enrolled patients will be summarized. Mean, standard deviation, median, and range will be used for continuous variables such as age. Absolute and relative frequencies will be used to describe categorical variables such as gender and ECOG PS. The proportion of patients receiving different chemotherapy regimens will be reported. Median, minimum, and maximum values will be used to summarize the number of weeks of each treatment and the total number of weeks of treatment.

9.3.3 Efficacy Analysis

Complete response rate of high-risk disease, defined by the absence of high-risk disease characteristics like T1, high-grade disease, CIS, or progressive disease;

Complete response rate of any disease, defined as the absence of any tumor evidence and progressive disease;

Duration of complete response of high-risk disease (measured from the initial evidence of complete response to high-risk disease recurrence or progression);

Duration of complete response of any disease (measured from the initial evidence of complete response to the emergence of any bladder tumor evidence).

9.3.4 Safety Analysis

For safety variables, AEs, SAEs, and their outcomes will be summarized and described by system and preferred terms. For adverse events, laboratory tests, vital signs, electrocardiograms, etc., the cumulative incidence of adverse events, adverse events leading to withdrawal from the study, adverse events leading to death, and serious adverse events by cycle will be summarized; regarding the severity of adverse events: if the same subject experiences the same adverse event multiple times, the most severe one will be included in the analysis; if the same subject experiences different adverse events, the most severe adverse event will be included in the analysis.

10 Ethics

This study will be conducted in full compliance with the Guidelines for Good Pharmacoepidemiology Practices (GPP) issued by the International Society for Pharmacoepidemiology, the relevant requirements of China's Good Clinical Practice (GCP), and Chinese laws and regulations.

10.1 Ethics Committee (EC)

Investigators shall submit relevant documents as required, including the study protocol, informed consent form, subject recruitment materials or advertisements, and other documents required by regulations, to the Ethics Committee (EC) for review and approval.

10.2 Ethics of the Study

The study process and the acquisition of informed consent shall comply with the Declaration of Helsinki, relevant GCP requirements, and Chinese laws and regulations related to drug and data protection.

GCP provides globally recognized quality standards that are scientifically and ethically sound for the design, conduct, documentation, and reporting of clinical studies involving human subjects. This study will be conducted in accordance with GCP and relevant national regulations, and will adhere to the relevant ethical principles in the Declaration of Helsinki to protect the rights, safety, and health of subjects.

10.3 Subject Information and Informed Consent

Before the start of any study procedure, the potential risks and benefits of the study shall be explained to potential subjects using the Informed Consent Form (ICF), and the language of the informed consent shall be simple and easy to understand. The ICF statement shall clearly state that informed consent is voluntarily signed, specify the potential risks and benefits of participating in the study, and that subjects may withdraw from the study at any time. Investigators may only enroll subjects after fully explaining the detailed content of the study, satisfactorily answering the subjects' questions, allowing sufficient time for consideration, and obtaining written consent from the subjects or their legal representatives. All signed informed consent forms must be kept in the investigator's documents or subject folders.

Investigators are responsible for explaining the content of the informed consent to subjects and obtaining the signed and dated informed consent form from the subjects or their legal representatives before the start of the study. After signing, the investigator shall provide the subject with a copy of the signed informed consent form. Investigators shall document the process of obtaining informed consent in the original study documents.

10.4 Protection of Subject Data

The ICF contains (or in some cases, together with a separate document) information related to data protection and privacy protection.

Preventive measures shall be taken to ensure the confidentiality of documents and prevent the identification of subjects' identities. However, in special circumstances, certain personnel may have access to a subject's genetic data and personal identification code. For example, in the event of a medical emergency, the attending physician or investigator may be aware of the subject's identification code and have access to the subject's genetic data. In addition, relevant regulatory authorities may require access to relevant documents.

11 Study Management

11.1 Data Processing and Record Keeping

Documents in the clinical trial (protocol and protocol amendments, completed CRFs, signed ICFs, etc.) shall be stored and managed in accordance with GCP requirements. The study center shall retain these documents for 5 years after the completion of the study. Study documents shall be properly stored to facilitate future access or data traceability. No study documents may be destroyed without the written permission of the investigator.

11.2 Protocol Amendment

All amendments to the protocol shall be kept as protocol supplements. Any modifications to the protocol shall be submitted to the Ethics Committee for approval or filing in accordance with the Ethics Committee's regulations.
