# Supplementary material for: Disitamab Vedotin Plus Tislelizumab in Transurethrally Unresectable, ERBB2-Positive, Non–Muscle-Invasive Bladder Cancer: A Nonrandomized Clinical Trial
Source: JAMA Oncol. 2026 Oct 8:e263909. Online ahead of print. doi: 10.1001/jamaoncol.2026.3909 (PMC13650702; doi:10.1001/jamaoncol.2026.3909)
Supplement: Supplementary file 2 [file jamaoncol-e263909-s002.pdf]

Supplementary Online Content

Qie Y, Jia K, Huang S, et al. Disitamab vedotin plus tislelizumab in transurethrally unresectable, *ERBB2*-positive, non–muscle-invasive bladder cancer: a nonrandomized clinical trial. *JAMA Oncol*. Published online October 8, 2026. doi:10.1001/jamaoncol.2026.3909

eFigure 1. Forest Plot of CR Rates for High-Risk Disease Across Different Subgroups

eFigure 2. DOR Among the 17 Patients Who Achieved an Initial CR of Any Disease

eFigure 3. DOR by *ERBB2* Expression Status

eFigure 4. PFS and OS by *ERBB2* Expression Status

eFigure 5. *ERBB2* Expression Dynamics in Noncomplete Responders After Treatment

eTable 1. CR Rate of Any Disease in Efficacy Assessment (n=26)

eTable 2. DV-Related Adverse Events (n=28)

eTable 3. Immune-Mediated Adverse Events (n=28)

eTable 4. Pathological Staging at Time of Radical Cystectomy in Patients Who Discontinued Investigational Treatment by Maximum T Stage (n=4)

This supplementary material has been provided by the authors to give readers additional information about their work.

© 2026 Qie Y et al. *JAMA Oncology*.

Figure S1. Forest plot of CR rates for high-risk disease across different subgroups.

Characteristics	n/N	CRR(95%CI)	P value
Overall	19/26	0.731(0.55-0.91)	
Sex, n (%)			0.54
Male	16/23	0.696(0.49-0.90)	
Female	3/3	1(1-1)	
Age, years, n (%)			0.41
≤70 years	7/11	0.636(0.30-0.98)	
>70 years	12/15	0.80(0.57-1)	
Smoking status, n (%)			0.19
Current/former smoker	5/9	0.556(0.15-0.96)	
Nonsmoker	14/17	0.824(0.62-1)	
ECOG performance status, n (%)			0.11
ECOG 0	17/21	0.81(0.63-0.99)	
ECOG 1	2/5	0.40(0-1)	
HER-2 status, n (%)			0.67
2+	11/14	0.786(0.54-1)	
3+	8/12	0.667(0.35-0.98)	
Tumor stage, n (%)			0.21
T1HG with CIS	4/8	0.50(0.5-0.95)	
CIS	2/2	1(1-1)	
T1HG alone	13/16	0.812(0.60-1)	
Patient type, n (%)			0.10
Primary tumor	17/21	0.81(0.63-0.99)	
Recurrent tumor	2/5	0.40(0-1)	
Histology, n (%)			0.29
Mixed	2/4	0.50(0-1)	
Pure urothelial	17/22	0.773(0.58-0.96)	

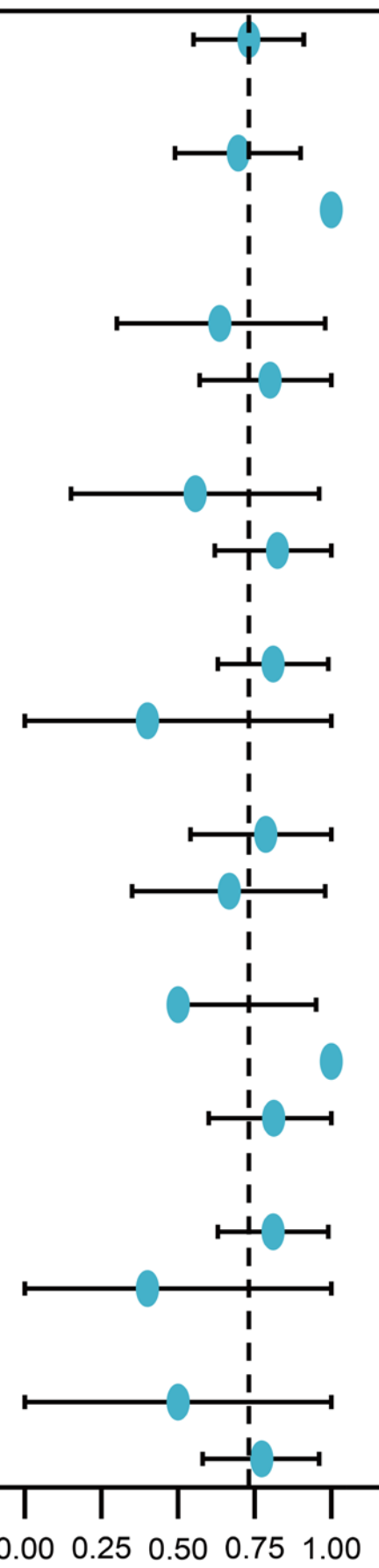

© 2026 Qie Y et al. *JAMA Oncology*.

Figure S2. DOR among the 17 patients who achieved an initial CR of any disease.

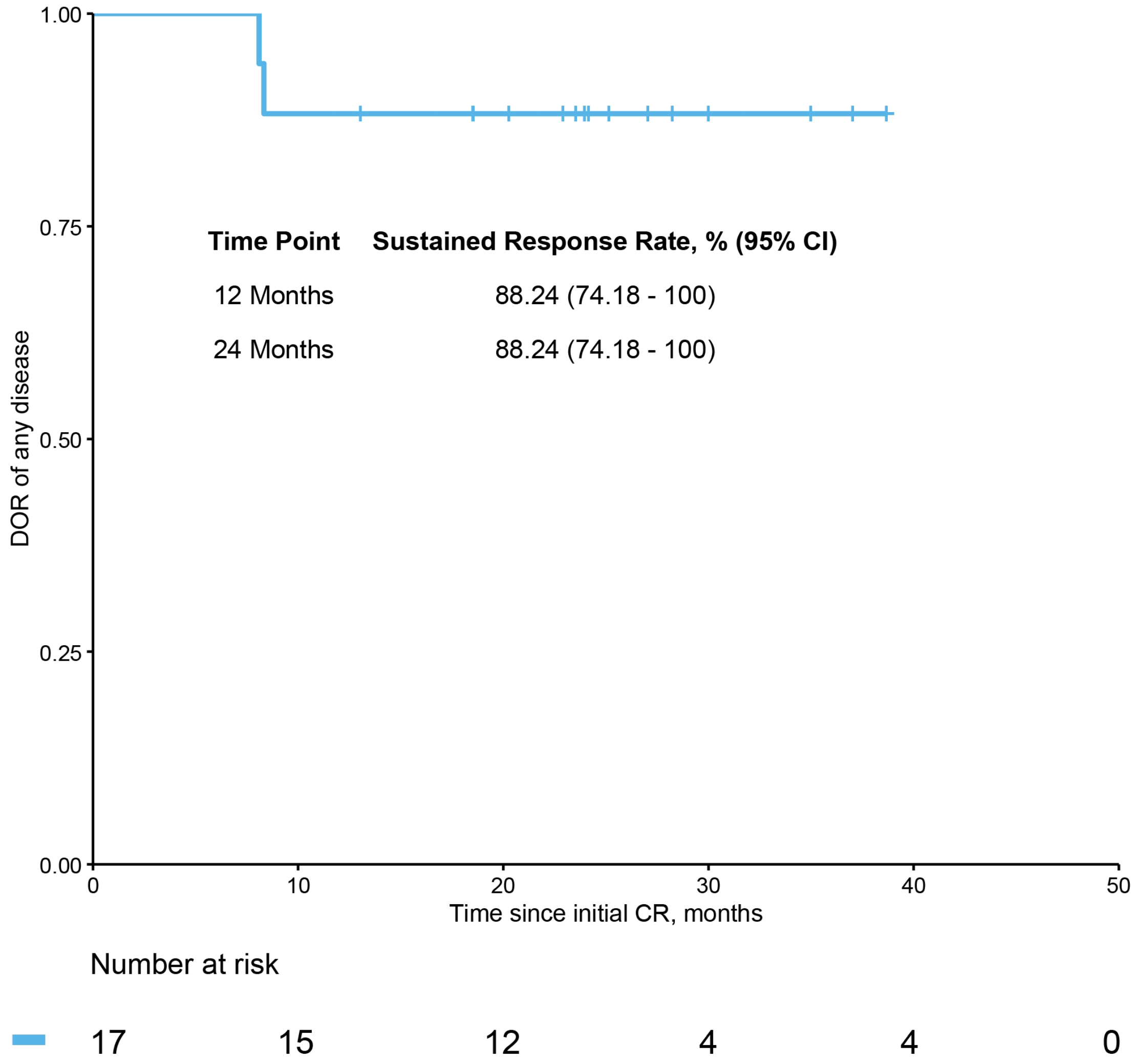

© 2026 Qie Y et al. *JAMA Oncology*.

Figure S3. DOR by *ERBB2* expression status.

A, DOR of high-risk disease, stratified by HER-2 expression (2+ vs 3+). B, DOR of any disease, stratified by HER-2 expression (2+ vs 3+). Tables show 12- and 24-month sustained response rates for each subgroup.

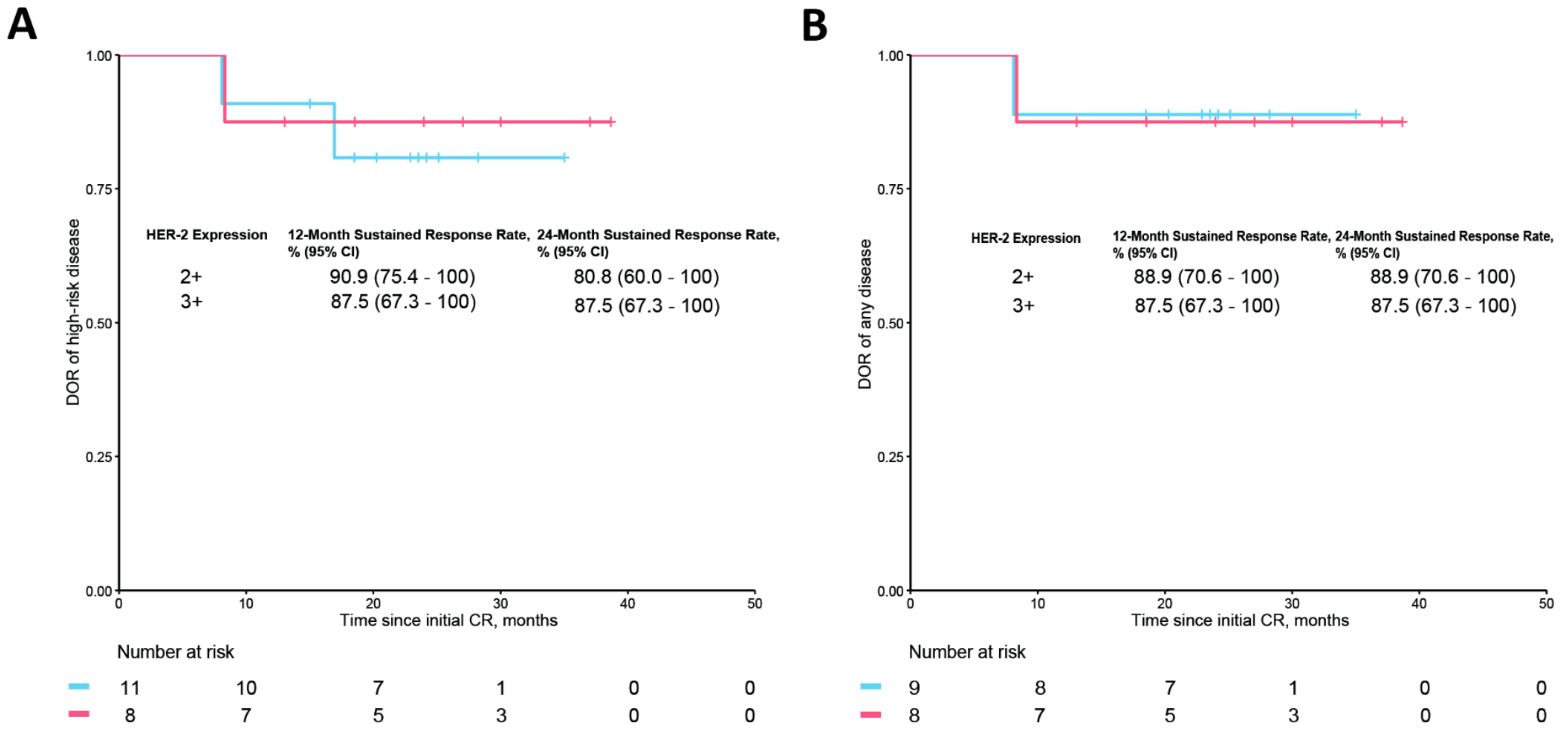

© 2026 Qie Y et al. *JAMA Oncology*.

Figure S4. PFS and OS by *ERBB2* expression status.

A, PFS, stratified by HER-2 expression (2+ vs 3+). B, OS, stratified by HER-2 expression (2+ vs 3+). Tables show 12- and 24-month survival rates for each subgroup.

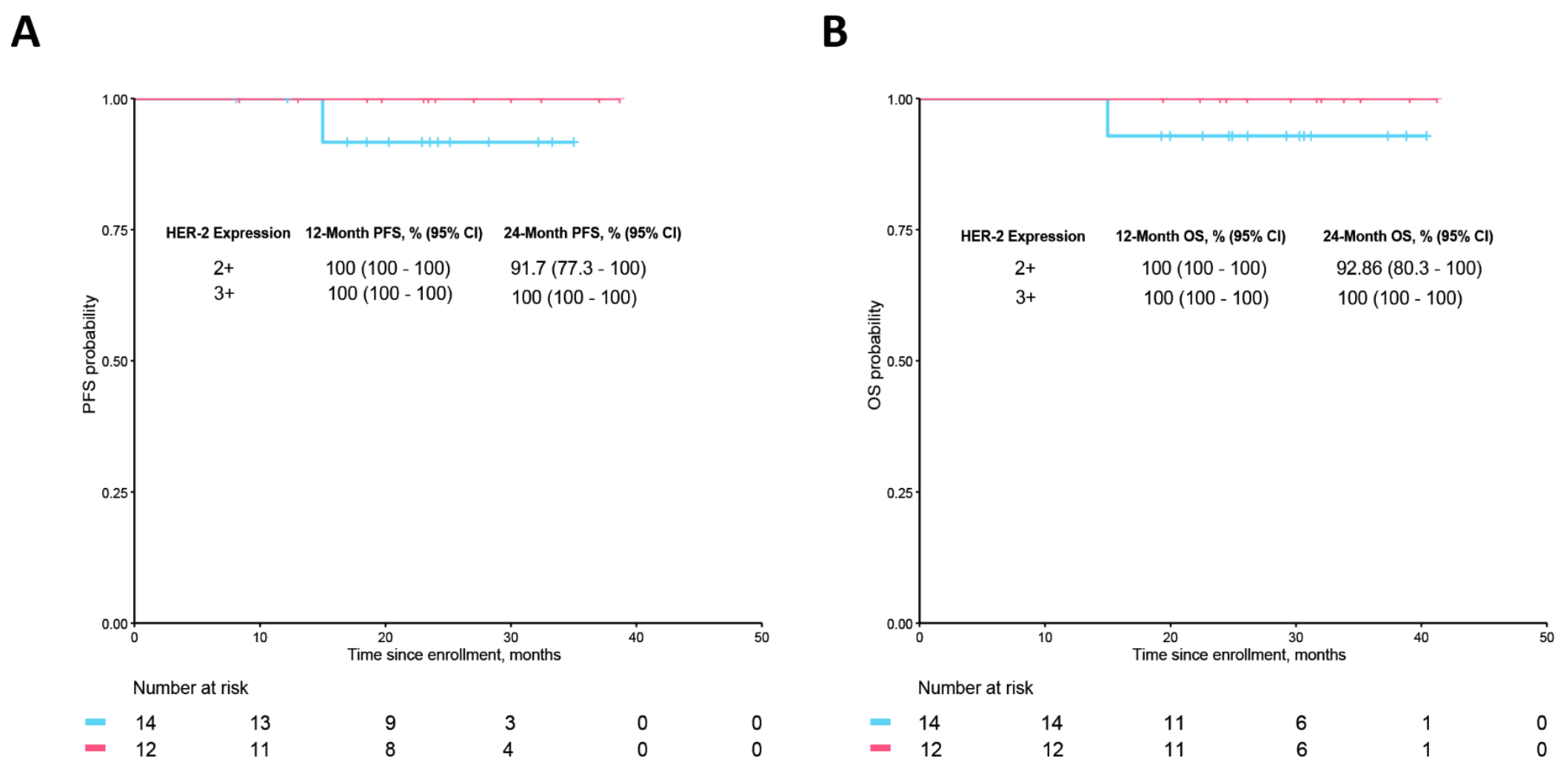

© 2026 Qie Y et al. *JAMA Oncology*.

Figure S5. *ERBB2* expression dynamics in non-complete responders after treatment.

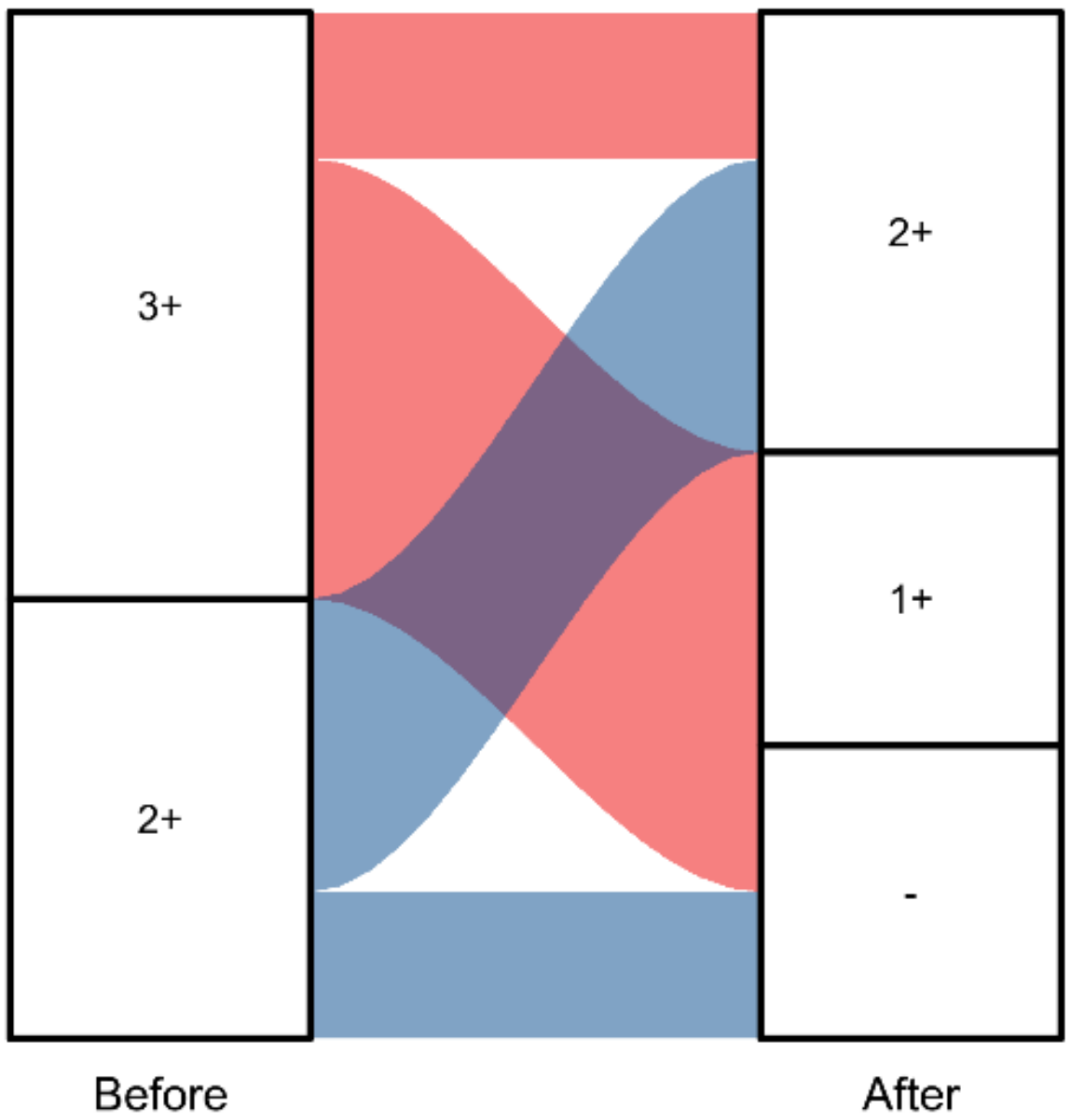

© 2026 Qie Y et al. *JAMA Oncology*.

Table S1. CR rate of any disease in efficacy assessment (n=26).

Endpoint	N (%)	95% CI
All patients (n=26)		
Complete response	17(65.4)	(95% CI: 45.8% to 85.0%)
Persistent disease[a]	9 (34.6)	(95% CI: 15.0% to 54.2%)
Her2 IHC 3+ (n=12)		
Complete response	8 (66.7)	(95% CI: 35.4% to 98.0%)
Persistent disease[a]	4 (33.3)	(95% CI: 2.0% to 64.6%)
Her2 IHC 2+ (n=14)		
Complete response	9 (64.3)	(95% CI: 35.6% to 93.0%)
Persistent disease[a]	5 (35.7)	(95% CI: 7.0% to 64.4%)

[a]Defined as the continued presence of non-muscle invasive bladder cancer (NMIBC) without evidence of progression in tumor grade or an increase in the depth of invasion.

© 2026 Qie Y et al. *JAMA Oncology*.

Table S2. DV-related adverse events (n=28).

AE	Patient, N (%)			
	**Any grade**	**Grade 1-2**	**Grade 3**	**Grade 4**
Any	22 (78.6)	22 (78.6)	0	0
Paresthesia	12 (42.9)	12 (42.9)	0	0
Alopecia	11 (39.3)	11 (39.3)	0	0
Anorexia	6 (21.4)	6 (21.4)	0	0
Nausea	4 (14.3)	4 (14.3)	0	0
Hepatic dysfunction	2 (7.1)	2 (7.1)	0	0
Constipation	2 (7.1)	2 (7.1)	0	0

© 2026 Qie Y et al. *JAMA Oncology*.

Table S3. Immune-mediated adverse events (n=28).

AE	Patient, N (%)			
	**Any grade**	**Grade 1-2**	**Grade 3**	**Grade 4**
Any	17 (60.7)	16 (57.1)	4 (14.3)	1 (3.6)
Pruritus	10 (35.7)	10 (35.7)	0	0
Rash	7 (25)	6 (21.4)	1(3.6)	0
Hypothyroidism	6 (21.4)	6 (21.4)	0	0
Fatigue	5 (17.9)	3 (10.7)	2(7.1)	0
Hypopituitarism	5 (17.9)	3 (10.7)	1(3.6)	1
Dry mouth	2 (7.1)	2 (7.1)	0	0
Edema	1 (3.6)	1 (3.6)	0	0
Myalgia	1 (3.6)	1 (3.6)	0	0
Immune-mediated colitis	1 (3.6)	1 (3.6)	0	0
Hyperglycemia	1 (3.6)	0	1(3.6)	0
Myocarditis	1 (3.6)	1 (3.6)	0	0
Increased creatine phosphokinase	1 (3.6)	1 (3.6)	0	0

© 2026 Qie Y et al. *JAMA Oncology*.

Table S4 Pathological staging at time of radical cystectomy in patients who discontinued investigational treatment by maximum T stage (n=4).

	**Patients, No.**	**N stage**	**Achieved initial complete response, No.**	**Number of investigational treatment doses, median (range)**	**Interval between last dose of investigational treatment and radical cystectomy, days, median (range)**
pTis	1	0	0	2	14
pT1	3	0	0	3 (3-3)	52 (21-103)

© 2026 Qie Y et al. *JAMA Oncology*.
