# Supplementary material for: Disitamab Vedotin Plus Tislelizumab in Transurethrally Unresectable, ERBB2-Positive, Non–Muscle-Invasive Bladder Cancer: A Nonrandomized Clinical Trial
Source: JAMA Oncol. 2026 Oct 8:e263909. Online ahead of print. doi: 10.1001/jamaoncol.2026.3909 (PMC13650702; doi:10.1001/jamaoncol.2026.3909)
Supplement: Supplementary file 3 [file jamaoncol-e263909-s003.pdf]

Data Sharing Statement

Qie. Disitamab Vedotin Plus Tislelizumab in Transurethrally Unresectable, ERBB2-Positive, Non–Muscle-Invasive Bladder Cancer. *JAMA Oncol.* Published October 08, 2026.
doi:10.1001/jamaoncol.2026.3909

Data
Additional Information: NCT05495724
Data available: Yes
Data types: Deidentified participant data
How to access data: Please contact huhailong@tmu.edu.cn
When available: With publication

Supporting Documents
Document types: Other (please specify)
Additional Information: Trial protocol
How to access documents: Please contact huhailong@tmu.edu.cn
When available: With publication

Additional Information
Who can access the data: Researchers whose proposed use of the data has been approved
Types of analyses: For any purpose
Mechanisms of data availability: After approval of a proposal
